# Supplementary material for: The Application of Optical Genome Mapping (OGM) in Severe Short Stature Caused by Duplication of 15q14q21.3
Source: Genes (Basel). 2023 Apr 29;14(5):1016. doi: 10.3390/genes14051016 (PMC10218401; doi:10.3390/genes14051016)

Table S1 The list of genes involved in duplication of 15q14q21.3.

Number	Gene/Region	Location (GRCh37)	Protein-Coding	OMIM	Function (NCBI Entrez Gene)
1	***FSIP1***	15:39892232-40075039	yes	615795	FSIP1 (Fibrous Sheath Interacting Protein 1) is a Protein Coding gene. Diseases associated with FSIP1 include Syndromic X-Linked Intellectual Disability Type 10 and Chromosome 3Q29 Duplication Syndrome.
2	***LOC105370941***	15:40075107-40077818	no	-	-
3	***GPR176***	15:40091223-40213093	yes	612183	Members of the G protein-coupled receptor family, such as GPR176, are cell surface receptors involved in responses to hormones, growth factors, and neurotransmitters
4	***LOC100505534***	15:40213211-40217100	no	-	-
5	***EIF2AK4***	15:40226325-40327797	yes	609280	This gene encodes a member of a family of kinases that phosphorylate the alpha subunit of eukaryotic translation initiation factor-2 (EIF2), resulting in the down-regulation of protein synthesis. The encoded protein responds to amino acid deprivation by binding uncharged transfer RNAs. It may also be activated by glucose deprivation and viral infection. Mutations in this gene have been found in individuals suffering from autosomal recessive pulmonary venoocclusive-disease-2.
6	***H3F3AP1***	15:40243027-40244090	no	-	-

7	***SRP14***	15:40327891-40331403	yes	600708	Component of the signal recognition particle (SRP) complex, a ribonucleoprotein complex that mediates the co-translational targeting of secretory and membrane proteins to the endoplasmic reticulum (ER) (PubMed:11089964). SRP9 together with SRP14 and the Alu portion of the SRP RNA, constitutes the elongation arrest domain of SRP (PubMed:11089964). The complex of SRP9 and SRP14 is required for SRP RNA binding (PubMed:11089964).
8	***SRP14-AS1***	15:40331512-40359710	no	-	-
9	***BMF***	15:40380091-40401085	yes	606266	May play a role in apoptosis. Isoform 1 seems to be the main initiator.
10	***MTND5P37***	15:40425972-40426744	no	-	-
11	***BUB1B***	15:40453210-40513337	yes	602860	Essential component of the mitotic checkpoint. Required for normal mitosis progression. The mitotic checkpoint delays anaphase until all chromosomes are properly attached to the mitotic spindle. One of its checkpoint functions is to inhibit the activity of the anaphase-promoting complex/cyclosome (APC/C) by blocking the binding of CDC20 to APC/C, independently of its kinase activity. The other is to monitor kinetochore activities that depend on the kinetochore motor CENPE. Required for kinetochore localization of CENPE. Negatively regulates PLK1 activity in

					interphase cells and suppresses centrosome amplification. Also implicated in triggering apoptosis in polyploid cells that exit aberrantly from mitotic arrest. May play a role for tumor suppression.
12	***BUB1B-PAK6***	15:40509629-40569688	no	-	-
13	***PAK6***	15:40531292-40569688	yes	608110	Serine/threonine protein kinase that plays a role in the regulation of gene transcription. The kinase activity is induced by various effectors including AR or MAP2K6/MAPKK6. Phosphorylates the DNA-binding domain of androgen receptor/AR and thereby inhibits AR-mediated transcription. Inhibits also ESR1-mediated transcription. May play a role in cytoskeleton regulation by interacting with IQGAP1. May protect cells from apoptosis through phosphorylation of BAD.
14	***C15orf56***	15:40542865-40545110	no	-	-
15	***ANKRD63***	15:40570573-40574787	yes	-	ANKRD63 (Ankyrin Repeat Domain 63) is a Protein Coding gene. An important paralog of this gene is CTTNBP2.This gene encodes a protein with six ankyrin repeats and several proline-rich regions. A similar gene in rat interacts with a central. CTTNBP2 (Cortactin Binding Protein 2) is a Protein Coding gene. Diseases associated with CTTNBP2 include Noonan Syndrome 12 and Brachial Plexus Neuritis. Gene Ontology (GO)

					annotations related to this gene include SH3 domain binding. An important paralog of this gene is TANC1. regulator of the actin cytoskeleton.
16	***PLCB2***	15:40580098-40600174	yes	604114	The production of the second messenger molecules diacylglycerol (DAG) and inositol 1,4,5-trisphosphate (IP3) is mediated by activated phosphatidylinositol-specific phospholipase C enzymes.
17	***RPLP0P10***	15:40604485-40605563	no	-	-
18	***INAFM2***	15:40616257-40618916	yes	-	Predicted to be integral component of membrane. [provided by Alliance of Genome Resources, Apr 2022]
19	***CCDC9B***	15:40623653-40633168	yes	-	Enables RNA binding activity. [provided by Alliance of Genome Resources, Apr 2022]
20	***RNA5SP392***	15:40623738-40623849	no	-	-
21	***PHGR1***	15:40643234-40648635	yes	-	PHGR1 (Proline, Histidine And Glycine Rich 1) is a Protein Coding gene. Diseases associated with PHGR1 include Diverticulitis Of Colon and Noonan Syndrome 3.
22	***DISP2***	15:40650436-40663257	yes	607503	This gene is one of two human homologs of a segment-polarity gene known as dispatched identified in Drosophila. The product of this gene may be required for normal Hedgehog (Hh) signalling during embryonic pattern formation. [provided by RefSeq, Jan 2017]
23	***KNSTRN***	15:40674922-40686489	yes	614718	Essential component of the mitotic spindle required

					for faithful chromosome segregation and progression into anaphase (PubMed:19667759). Promotes the metaphase-to-anaphase transition and is required for chromosome alignment, normal timing of sister chromatid segregation, and maintenance of spindle pole architecture (PubMed:19667759, PubMed:22110139). The astrin (SPAG5)-kinastrin (SKAP) complex promotes stable microtubule-kinetochore attachments (PubMed:21402792). Required for kinetochore oscillations and dynamics of microtubule plus-ends during live cell mitosis, possibly by forming a link between spindle microtubule plus-ends and mitotic chromosomes to achieve faithful cell division (PubMed:23035123). May be involved in UV-induced apoptosis via its interaction with PRPF19; however, these results need additional evidences (PubMed:24718257).
24	***IVD***	15:40697686-40713512	yes	607036	Catalyzes the conversion of isovaleryl-CoA/3-methylbutanoyl-CoA to 3-methylbut-2-enoyl-CoA as an intermediate step in the leucine (Leu) catabolic pathway (PubMed:7640268). To a lesser extent, is also able to catalyze the oxidation of other saturated short-chain acyl-CoA thioesters as pentanoyl-CoA, hexenoyl-CoA and butenoyl-CoA (PubMed:7640268).

25	***BAHD1***	15:40731920-40760441	yes	613880	Heterochromatin protein that acts as a transcription repressor and has the ability to promote the formation of large heterochromatic domains. May act by recruiting heterochromatin proteins such as CBX5 (HP1 alpha), HDAC5 and MBD1. Represses IGF2 expression by binding to its CpG-rich P3 promoter and recruiting heterochromatin proteins. At specific stages of Listeria infection, in complex with TRIM28, corepresses interferon-stimulated genes, including IFNL1, IFNL2 and IFNL3.
26	***CHST14***	15:40763160-40765357	yes	608429	Catalyzes the transfer of sulfate to position 4 of the N-acetylgalactosamine (GalNAc) residue of dermatan sulfate. Plays a pivotal role in the formation of 4-0-sulfated IdoA blocks in dermatan sulfate. Transfers sulfate to the C-4 hydroxyl of beta1,4-linked GalNAc that is substituted with an alpha-linked iduronic acid (IdoUA) at the C-3 hydroxyl. Transfers sulfate more efficiently to GalNAc residues in -IdoUA-GalNAc-IdoUA- than in -GlcUA-GalNAc-GlcUA-sequences. Has preference for partially desulfated dermatan sulfate. Addition of sulfate to GalNAc may occur immediately after epimerization of GlcUA to IdoUA. Appears to have an important role in the formation of the cerebellar neural network during postnatal brain development.

27	***RPL9P27***	15:40772850-40773549	no	-	-
28	***RNU6-516P***	15:40821769-40821841	no	-	-
29	***CCDC32***	15:40823540-40857256	yes	618941	Has a role in ciliogenesis (By similarity). Required for proper cephalic and left/right axis development (PubMed:32307552).
30	***MRPL42P5***	15:40824083-40824749	no	-	-
31	***RPUSD2***	15:40861492-40866905	yes	-	Pseudouridine synthase that catalyzes pseudouridylation of mRNAs.
32	***TRS-GCT4-2***	15:40886023-40886104	no	-	-
33	***KNL1***	15:40886447-40954881	yes	609173	Performs two crucial functions during mitosis: it is essential for spindle-assembly checkpoint signaling and for correct chromosome alignment. Required for attachment of the kinetochores to the spindle microtubules. Directly links BUB1 and BUB1B to kinetochores. Part of the MIS12 complex, which may be fundamental for kinetochore formation and proper chromosome segregation during mitosis. Acts in coordination with CENPK to recruit the NDC80 complex to the outer kinetochore.
34	***RAD51-AS1***	15:40985932-40987303	no	-	-
35	***RAD51***	15:40987327-41024356	yes	179617	Plays an important role in homologous strand exchange, a key step in DNA repair through homologous recombination (HR) (PubMed:18417535, PubMed:20348101, PubMed:12205100, PubMed:20231364, PubMed:23754376, PubMed:23509288,

					PubMed:28575658, PubMed:26681308). Binds to single-stranded DNA in an ATP-dependent manner to form nucleoprotein filaments which are essential for the homology search and strand exchange (PubMed:18417535, PubMed:20348101, PubMed:12205100, PubMed:20231364, PubMed:23754376, PubMed:23509288, PubMed:28575658, PubMed:26681308). Catalyzes the recognition of homology and strand exchange between homologous DNA partners to form a joint molecule between a processed DNA break and the repair template (PubMed:18417535, PubMed:20348101, PubMed:12205100, PubMed:20231364, PubMed:23754376, PubMed:23509288, PubMed:28575658, PubMed:26681308). Recruited to resolve stalled replication forks during replication stress (PubMed:27797818, PubMed:31844045). Part of a PALB2-scaffolded HR complex containing BRCA2 and RAD51C and which is thought to play a role in DNA repair by HR (PubMed:24141787, PubMed:12442171). Plays a role in regulating mitochondrial DNA copy number under conditions of oxidative stress in the presence of RAD51C and XRCC3 (PubMed:20413593). Also involved in interstrand cross-link repair (PubMed:26253028).

36	***RMDN3***	15:41028082-41047534	yes	611873	Involved in cellular calcium homeostasis regulation. May participate in differentiation and apoptosis of keratinocytes. Overexpression induces apoptosis.
37	***SUMO2P15***	15:41052795-41053072	no	-	-
38	***GCHFR***	15:41056285-41059911	yes	602437	Mediates tetrahydrobiopterin inhibition of GTP cyclohydrolase 1. This inhibition is reversed by L-phenylalanine.
39	***DNAJC17***	15:41060067-41099676	yes	616844	May negatively affect PAX8-induced thyroglobulin/TG transcription.
40	***C15orf62***	15:41062159-41064647	yes	-	Predicted to be involved in Rho protein signal transduction; positive regulation of cellular component biogenesis; and regulation of cell shape. Predicted to be located in mitochondrion. Predicted to be active in cytoplasm; cytoskeleton; and plasma membrane. [provided by Alliance of Genome Resources, Apr 2022]
41	***ZFYVE19***	15:41099274-41106767	yes	619635	Key regulator of abscission step in cytokinesis: part of the cytokinesis checkpoint, a process required to delay abscission to prevent both premature resolution of intercellular chromosome bridges and accumulation of DNA damage. Together with CHMP4C, required to retain abscission-competent VPS4 (VPS4A and/or VPS4B) at the midbody ring until abscission checkpoint signaling is terminated at late cytokinesis. Deactivation of AURKB results

					in dephosphorylation of CHMP4C followed by its dissociation from ZFYVE19/ANCHR and VPS4 and subsequent abscission.
42	***PPP1R14D***	15:41107643-41120907	yes	613256	Inhibitor of PPP1CA. Has inhibitory activity only when phosphorylated, creating a molecular switch for regulating the phosphorylation status of PPP1CA substrates and smooth muscle contraction.
43	***SPINT1***	15:41136246-41149853	yes	605123	Inhibitor of HGF activator. Also acts as an inhibitor of matriptase (ST14).
44	***RHOV***	15:41164412-41166487	yes	-	Plays a role in the control of the actin cytoskeleton via activation of the JNK pathway.
45	***VPS18***	15:41186628-41196173	yes	608551	Plays a role in vesicle-mediated protein trafficking to lysosomal compartments including the endocytic membrane transport and autophagic pathways. Believed to act as a core component of the putative HOPS and CORVET endosomal tethering complexes which are proposed to be involved in the Rab5-to-Rab7 endosome conversion probably implicating MON1A/B, and via binding SNAREs and SNARE complexes to mediate tethering and docking events during SNARE-mediated membrane fusion. The HOPS complex is proposed to be recruited to Rab7 on the late endosomal membrane and to regulate late endocytic, phagocytic and autophagic traffic towards lysosomes. The CORVET complex is proposed to

					function as a Rab5 effector to mediate early endosome fusion probably in specific endosome subpopulations (PubMed:11382755, PubMed:23351085, PubMed:24554770, PubMed:25783203). Required for fusion of endosomes and autophagosomes with lysosomes (PubMed:25783203). Involved in dendrite development of Pukinje cells (By similarity).
46	***LOC105370943***	15:41199007-41201549	no	-	-
47	***DLL4***	15:41221531-41231258	yes	605185	Involved in the Notch signaling pathway as Notch ligand (PubMed:11134954). Activates NOTCH1 and NOTCH4. Involved in angiogenesis; negatively regulates endothelial cell proliferation and migration and angiogenic sprouting (PubMed:20616313). Essential for retinal progenitor proliferation. Required for suppressing rod fates in late retinal progenitors as well as for proper generation of other retinal cell types (By similarity). During spinal cord neurogenesis, inhibits V2a interneuron fate (PubMed:17728344).
48	***ISCA1P4***	15:41240685-41241508	no	-	-
49	***CHAC1***	15:41245347-41248717	yes	614587	Catalyzes the cleavage of glutathione into 5-oxo-L-proline and a Cys-Gly dipeptide. Acts specifically on glutathione, but not on other gamma-glutamyl peptides (PubMed:27913623). Glutathione depletion is an important factor for apoptosis

					initiation and execution. Acts as a pro-apoptotic component of the unfolded protein response pathway by mediating the pro-apoptotic effects of the ATF4-ATF3-DDIT3/CHOP cascade (PubMed:19109178). Negative regulator of Notch signaling pathway involved in embryonic neurogenesis: acts by inhibiting Notch cleavage by furin, maintaining Notch in an immature inactive form, thereby promoting neurogenesis in embryos (PubMed:22445366).
50	***INO80***	15:41271078-41408444	yes	610169	ATPase component of the chromatin remodeling INO80 complex which is involved in transcriptional regulation, DNA replication and DNA repair (PubMed:16230350, PubMed:16298340, PubMed:17721549, PubMed:20855601, PubMed:20237820). Binds DNA (PubMed:16298340, PubMed:21303910). As part of the INO80 complex, remodels chromatin by shifting nucleosomes (PubMed:16230350, PubMed:21303910). Regulates transcription upon recruitment by YY1 to YY1-activated genes, where it acts as an essential coactivator (PubMed:17721549). Involved in UV-damage excision DNA repair (PubMed:20855601). The contribution to DNA double-strand break repair appears to be largely indirect through

					transcriptional regulation (PubMed:20687897). Involved in DNA replication (PubMed:20237820). Required for microtubule assembly during mitosis thereby regulating chromosome segregation cycle (PubMed:20237820).
51	***CYCSP2***	15:41401063-41401375	no	-	-
52	***FAM92A1P1***	15:41455322-41456895	no	-	-
53	***EXD1***	15:41474926-41522955	yes	-	RNA-binding component of the PET complex, a multiprotein complex required for the processing of piRNAs during spermatogenesis. The piRNA metabolic process mediates the repression of transposable elements during meiosis by forming complexes composed of piRNAs and Piwi proteins and governs the methylation and subsequent repression of transposable elements, preventing their mobilization, which is essential for the germline integrity (By similarity). The PET complex is required during the secondary piRNAs metabolic process for the PIWIL2 slicing-triggered loading of PIWIL4 piRNAs. In the PET complex, EXD1 probably acts as an RNA adapter. EXD1 is an inactive exonuclease (By similarity).
54	***RN7SL497P***	15:41484163-41484463	no	-	-
55	***CHP1***	15:41523437-41574085	yes	606988	Calcium-binding protein involved in different processes such as regulation of vesicular trafficking, plasma membrane Na(+)/H(+)

					exchanger and gene transcription. Involved in the constitutive exocytic membrane traffic. Mediates the association between microtubules and membrane-bound organelles of the endoplasmic reticulum and Golgi apparatus and is also required for the targeting and fusion of transcytotic vesicles (TCV) with the plasma membrane. Functions as an integral cofactor in cell pH regulation by controlling plasma membrane-type Na(+)/H(+) exchange activity. Affects the pH sensitivity of SLC9A1/NHE1 by increasing its sensitivity at acidic pH. Required for the stabilization and localization of SLC9A1/NHE1 at the plasma membrane. Inhibits serum- and GTPase-stimulated Na(+)/H(+) exchange. Plays a role as an inhibitor of ribosomal RNA transcription by repressing the nucleolar UBF1 transcriptional activity. May sequester UBF1 in the nucleoplasm and limit its translocation to the nucleolus. Associates to the ribosomal gene promoter. Acts as a negative regulator of the calcineurin/NFAT signaling pathway. Inhibits NFAT nuclear translocation and transcriptional activity by suppressing the calcium-dependent calcineurin phosphatase activity. Also negatively regulates the kinase activity of the apoptosis-induced kinase STK17B. Inhibits both

					STK17B auto- and substrate-phosphorylations in a calcium-dependent manner.
56	***OIP5-AS1***	15:41576201-41591795	no	-	-
57	***OIP5***	15:41601466-41624819	yes	606020	Required for recruitment of CENPA to centromeres and normal chromosome segregation during mitosis.
58	***NUSAP1***	15:41624892-41673248	yes	612818	Microtubule-associated protein with the capacity to bundle and stabilize microtubules (By similarity). May associate with chromosomes and promote the organization of mitotic spindle microtubules around them.
59	***NDUFAF1***	15:41679547-41694658	yes	606934	As part of the MCIA complex, involved in the assembly of the mitochondrial complex I.
60	***RTF1***	15:41709302-41775761	yes	611633	Component of the PAF1 complex (PAF1C) which has multiple functions during transcription by RNA polymerase II and is implicated in regulation of development and maintenance of embryonic stem cell pluripotency. PAF1C associates with RNA polymerase II through interaction with POLR2A CTD non-phosphorylated and 'Ser-2'- and 'Ser-5'-phosphorylated forms and is involved in transcriptional elongation, acting both independently and synergistically with TCEA1 and in cooperation with the DSIF complex and HTATSF1. PAF1C is required for transcription of Hox and Wnt target genes. PAF1C is involved in

					hematopoiesis and stimulates transcriptional activity of KMT2A/MLL1; it promotes leukemogenesis through association with KMT2A/MLL1-rearranged oncoproteins, such as KMT2A/MLL1-MLLT3/AF9 and KMT2A/MLL1-MLLT1/ENL. PAF1C is involved in histone modifications such as ubiquitination of histone H2B and methylation on histone H3 'Lys-4' (H3K4me3). PAF1C recruits the RNF20/40 E3 ubiquitin-protein ligase complex and the E2 enzyme UBE2A or UBE2B to chromatin which mediate monoubiquitination of 'Lys-120' of histone H2B (H2BK120ub1); UB2A/B-mediated H2B ubiquitination is proposed to be coupled to transcription. PAF1C is involved in mRNA 3' end formation probably through association with cleavage and poly(A) factors. In case of infection by influenza A strain H3N2, PAF1C associates with viral NS1 protein, thereby regulating gene transcription. Binds single-stranded DNA. Required for maximal induction of heat-shock genes. Required for the trimethylation of histone H3 'Lys-4' (H3K4me3) on genes involved in stem cell pluripotency; this function is synergistic with CXXC1 indicative for an involvement of a SET1 complex (By similarity).

61	***ITPKA***	15:41786056-41795757	yes	147521	Catalyzes the phosphorylation of 1D-myo-inositol 1,4,5-trisphosphate (InsP3) into 1D-myo-inositol 1,3,4,5-tetrakisphosphate and participates to the regulation of calcium homeostasis.
62	***LTK***	15:41795840-41806085	yes	151520	Receptor with a tyrosine-protein kinase activity (PubMed:10445845, PubMed:20548102, PubMed:30061385). Following activation by ALKAL1 or ALKAL2 ligands at the cell surface, transduces an extracellular signal into an intracellular response (PubMed:30061385, PubMed:34646012). Ligand-binding to the extracellular domain induces tyrosine kinase activation, leading to activation of the mitogen-activated protein kinase (MAPK) pathway (PubMed:20548102). Phosphorylates almost exclusively at the first tyrosine of the Y-x-x-x-Y-Y motif (By similarity). The exact function of this protein is not known; studies with chimeric proteins demonstrate its ability to promote growth and specifically neurite outgrowth, and cell survival (PubMed:9223670, PubMed:18849880). Involved in regulation of the secretory pathway involving endoplasmic reticulum (ER) export sites (ERESs) and ER to Golgi transport (PubMed:20548102).
63	***RPAP1***	15:41809374-41836475	yes	611475	Forms an interface between the RNA polymerase II enzyme and chaperone/scaffolding protein,

					suggesting that it is required to connect RNA polymerase II to regulators of protein complex formation. Required for interaction of the RNA polymerase II complex with acetylated histone H3.
64	***ELOCP2***	15:41849133-41849861	no	-	-
65	***TYRO3***	15:41851220-41871536	yes	600341	(Microbial infection) Acts as a receptor for Ebolavirus, possibly through GAS6 binding to phosphatidyl-serine at the surface of virion envelope.‖(Microbial infection) Acts as a receptor for lassa virus and lymphocytic choriomeningitis virus, possibly through GAS6 binding to phosphatidyl-serine at the surface of virion envelope.‖Receptor tyrosine kinase that transduces signals from the extracellular matrix into the cytoplasm by binding to several ligands including TULP1 or GAS6. Regulates many physiological processes including cell survival, migration and differentiation. Ligand binding at the cell surface induces dimerization and autophosphorylation of TYRO3 on its intracellular domain that provides docking sites for downstream signaling molecules. Following activation by ligand, interacts with PIK3R1 and thereby enhances PI3-kinase activity. Activates the AKT survival pathway, including nuclear translocation of NF-kappa-B and up-regulation of transcription of NF-kappa-B-

					regulated genes. TYRO3 signaling plays a role in various processes such as neuron protection from excitotoxic injury, platelet aggregation and cytoskeleton reorganization. Also plays an important role in inhibition of Toll-like receptors (TLRs)-mediated innate immune response by activating STAT1, which selectively induces production of suppressors of cytokine signaling SOCS1 and SOCS3.
66	***MGA***	15:41952610-42062141	yes	616061	Functions as a dual-specificity transcription factor, regulating the expression of both MAX-network and T-box family target genes. Functions as a repressor or an activator. Binds to 5'-AATTTCACACCTAGGTGTGAAATT-3' core sequence and seems to regulate MYC-MAX target genes. Suppresses transcriptional activation by MYC and inhibits MYC-dependent cell transformation. Function activated by heterodimerization with MAX. This heterodimerization serves the dual function of both generating an E-box-binding heterodimer and simultaneously blocking interaction of a corepressor (By similarity).
67	***MIR626***	15:41983783-41983876	no	-	-
68	***MAPKBP1***	15:42066632-42120053	yes	616786	Negative regulator of NOD2 function. It down-regulates NOD2-induced processes such as

					activation of NF-kappa-B signaling, IL8 secretion and antibacterial response (PubMed:22700971). Involved in JNK signaling pathway (By similarity).
69	***JMJD7***	15:42120283-42129785	yes	-	Bifunctional enzyme that acts both as an endopeptidase and 2-oxoglutarate-dependent monooxygenase (PubMed:28847961, PubMed:29915238). Endopeptidase that cleaves histones N-terminal tails at the carboxyl side of methylated arginine or lysine residues, to generate 'tailless nucleosomes', which may trigger transcription elongation (PubMed:28847961). Preferentially recognizes and cleaves monomethylated and dimethylated arginine residues of histones H2, H3 and H4 (PubMed:28847961). After initial cleavage, continues to digest histones tails via its aminopeptidase activity (PubMed:28847961). Additionally, may play a role in protein biosynthesis by modifying the translation machinery (PubMed:29915238). Acts as Fe(2+) and 2-oxoglutarate-dependent monooxygenase, catalyzing (S)-stereospecific hydroxylation at C-3 of 'Lys-22' of DRG1 and 'Lys-21' of DRG2 translation factors (TRAFAC), promoting their interaction with ribonucleic acids (RNA) (PubMed:29915238).

70	***JMJD7-PLA2G4B***	15:42120283-42140346	no	-	-
71	***PLA2G4B***	15:42131011-42140346	yes	606088	[Isoform 3]: Calcium-dependent phospholipase A2 and lysophospholipase. Cleaves the ester bond of the fatty acyl group attached to the sn-2 position of phosphatidylethanolamines, producing lysophospholipids that may be used in deacylation-reacylation cycles. Hydrolyzes lysophosphatidylcholines with low efficiency but is inefficient toward phosphatidylcholines.||[Isoform 5]: Calcium-dependent phospholipase A1 and A2 and lysophospholipase. Cleaves the ester bond of the fatty acyl group attached to the sn-1 or sn-2 position of diacyl phospholipids (phospholipase A1 and A2 activity, respectively), producing lysophospholipids that may be used in deacylation-reacylation cycles. Can further hydrolyze lysophospholipids enabling complete deacylation. Has no activity toward alkylacyl phospholipids.||Calcium-dependent phospholipase A1 and A2 and lysophospholipase that may play a role in membrane phospholipid remodeling.
72	***SPTBN5***	15:42140344-42186275	yes	605916	Enables several functions, including cytoskeletal protein binding activity; dynein intermediate chain binding activity; and identical protein binding activity. Acts upstream of or within Golgi organization and lysosomal transport. Located in

					cytoplasm; photoreceptor connecting cilium; and photoreceptor disc membrane. [provided by Alliance of Genome Resources, Apr 2022]
73	***RNA5SP393***	15:42144111-42144227	no	-	-
74	***MIR4310***	15:42158693-42158749	no	-	-
75	***LOC105370792***	15:42184975-42191013	no	-	-
76	***LOC100289090***	15:42186325-42187530	no	-	-
77	***EHD4***	15:42191638-42264755	yes	605892	ATP- and membrane-binding protein that probably controls membrane reorganization/tubulation upon ATP hydrolysis. Plays a role in early endosomal transport.
78	***EHD4-AS1***	15:42213615-42221484	no	-	-
79	***PLA2G4E-AS1***	15:42264961-42291292	no	-	-
80	***PLA2G4E***	15:42273780-42342901	yes	-	Calcium-dependent N-acyltransferase involved in the biosynthesis of N-acyl ethanolamines (NAEs) in the brain (PubMed:29447909). Transfers the sn-1 fatty acyl chain of phosphatidylcholine (fatty acyl donor) to the amine group of phosphatidylethanolamine (fatty acyl acceptor) to generate N-acyl phosphatidylethanolamine (NAPE). Similarly can use plasmenylethanolamine as a fatty acyl acceptor to form N-acyl plasmenylethanolamine (N-Acyl-PlsEt). Both NAPE and N-Acyl-PlsEt can serve as precursors of bioactive NAEs like N-arachidonoyl phosphatidylethanolamine also called anandamide

					(PubMed:29447909, PubMed:30517655). Has weak phospholipase A2 and lysophospholipase activities (By similarity). Regulates intracellular membrane trafficking that requires modulation of membrane curvature as it occurs by enrichment in lysophospholipids. Promotes tubule formation involved in clathrin-independent endocytotic trafficking and cargo recycling (By similarity).
81	***PLA2G4D***	15:42359881-42386752	yes	612864	Calcium-dependent phospholipase A2 that selectively hydrolyzes glycerophospholipids in the sn-2 position (PubMed:14709560). Has a preference for linoleic acid at the sn-2 position (PubMed:14709560).
82	***PLA2G4F***	15:42433332-42448839	yes	-	Has calcium-dependent phospholipase and lysophospholipase activities with a potential role in membrane lipid remodeling and biosynthesis of lipid mediators (PubMed:29158256). Preferentially hydrolyzes the ester bond of the fatty acyl group attached at sn-2 position of phospholipids (phospholipase A2 activity) (PubMed:29158256). Selectively hydrolyzes sn-2 arachidonoyl group from membrane phospholipids, providing the precursor for eicosanoid biosynthesis (PubMed:29158256). In myocardial mitochondria, plays a major role in arachidonate release that is metabolically channeled to the formation of

					cardioprotective eicosanoids, epoxyeicosatrienoates (EETs) (PubMed:29158256).
83	***VPS39***	15:42450899-42500524	yes	612188	Plays a role in vesicle-mediated protein trafficking to lysosomal compartments including the endocytic membrane transport and autophagic pathways. Acts as a component of the putative HOPS endosomal tethering complex which is proposed to be involved in the Rab5-to-Rab7 endosome conversion probably implicating MON1A/B, and via binding SNAREs and SNARE complexes to mediate tethering and docking events during SNARE-mediated membrane fusion. The HOPS complex is proposed to be recruited to Rab7 on the late endosomal membrane and to regulate late endocytic, phagocytic and autophagic traffic towards lysosomes (PubMed:23351085). Involved in homotypic vesicle fusions between late endosomes and in heterotypic fusions between late endosomes and lysosomes (PubMed:11448994, PubMed:23351085, PubMed:23167963). Required for fusion of endosomes and autophagosomes with lysosomes (PubMed:25783203).||Regulator of TGF-beta/activin signaling, inhibiting SMAD3- and activating SMAD2-dependent transcription. Acts by interfering with SMAD3/SMAD4 complex

					formation, this would lead to inhibition of SMAD3-dependent transcription and relieve SMAD3 inhibition of SMAD2-dependent promoters, thus increasing SMAD2-dependent transcription. Does not affect TGF-beta-induced SMAD2 or SMAD3 phosphorylation, nor SMAD2/SMAD4 complex formation.
84	***MIR627***	15:42491768-42491864	no	-	-
85	***TMEM87A***	15:42502650-42565782	yes	-	May be involved in retrograde transport from endosomes to the trans-Golgi network (TGN).
86	***GANC***	15:42565856-42645864	yes	104180	Has alpha-glucosidase activity.
87	***BNIP3P5***	15:42606185-42606877	no	-	-
88	***CAPN3***	15:42651698-42704515	yes	114240	Calcium-regulated non-lysosomal thiol-protease. Proteolytically cleaves CTBP1 at 'His-409'. Mediates, with UTP25, the proteasome-independent degradation of p53/TP53 (PubMed:23357851, PubMed:27657329).
89	***ZNF106***	15:42704635-42783395	yes	-	RNA-binding protein. Specifically binds to 5'-GGGGCC-3' sequence repeats in RNA. Essential for maintenance of peripheral motor neuron and skeletal muscle function. Required for normal expression and/or alternative splicing of a number of genes in spinal cord and skeletal muscle, including the neurite outgrowth inhibitor RTN4. Also contributes to normal mitochondrial respiratory function in motor neurons, via an

					unknown mechanism.
90	***RNU6-188P***	15:42719789-42719895	no	-	-
91	***SNAP23***	15:42787504-42825259	yes	602534	Essential component of the high affinity receptor for the general membrane fusion machinery and an important regulator of transport vesicle docking and fusion.
92	***LOC100505769***	15:42817139-42819162	no	-	-
93	***LRRC57***	15:42834720-42841002	yes	-	Located in extracellular exosome. [provided by Alliance of Genome Resources, Apr 2022]
94	***HAUS2***	15:42841011-42862192	yes	613429	Contributes to mitotic spindle assembly, maintenance of centrosome integrity and completion of cytokinesis as part of the HAUS augmin-like complex.
95	***MYL12BP1***	15:42864022-42864820	no	-	-
96	***STARD9***	15:42867857-43013196	yes	614642	Microtubule-dependent motor protein required for spindle pole assembly during mitosis. Required to stabilize the pericentriolar material (PCM).
97	***EIF4EBP2P2***	15:42870830-42873908	no	-	-
98	***CDAN1***	15:43015760-43029417	yes	607465	May act as a negative regulator of ASF1 in chromatin assembly.
99	***TTBK2***	15:43036536-43213007	yes	611695	Serine/threonine kinase that acts as a key regulator of ciliogenesis: controls the initiation of ciliogenesis by binding to the distal end of the basal body and promoting the removal of CCP110, which caps the mother centriole, leading to the recruitment of IFT proteins, which build the ciliary axoneme.

					Has some substrate preference for proteins that are already phosphorylated on a Tyr residue at the +2 position relative to the phosphorylation site. Able to phosphorylate tau on serines in vitro (PubMed:23141541). Phosphorylates MPHOSPH9 which promotes its ubiquitination and proteasomal degradation, loss of MPHOSPH9 facilitates the removal of the CP110-CEP97 complex (a negative regulator of ciliogenesis) from the mother centrioles, promoting the initiation of ciliogenesis (PubMed:30375385).
100	***KRT8P50***	15:43092966-43094667	no	-	-
101	***LOC100505791***	15:43093161-43094604	no	-	-
102	***FDPSP4***	15:43231451-43232602	no	-	-
103	***UBR1***	15:43235095-43398286	yes	605981	E3 ubiquitin-protein ligase which is a component of the N-end rule pathway. Recognizes and binds to proteins bearing specific N-terminal residues that are destabilizing according to the N-end rule, leading to their ubiquitination and subsequent degradation. May be involved in pancreatic homeostasis. Binds leucine and is a negative regulator of the leucine-mTOR signaling pathway, thereby controlling cell growth.
104	***RPS3AP47***	15:43407853-43408715	no	-	-
105	***SPCS2P1***	15:43423217-43423885	no	-	-
106	***TMEM62***	15:43425313-43477342	yes	-	Predicted to enable hydrolase activity. Predicted to

					be integral component of membrane. [provided by Alliance of Genome Resources, Apr 2022]
107	***LOC100505831***	15:43430024-43430252	no	-	-
108	***FDPSP10***	15:43432291-43433465	no	-	-
109	***CCNDBP1***	15:43477466-43489375	yes	607089	May negatively regulate cell cycle progression. May act at least in part via inhibition of the cyclin-D1/CDK4 complex, thereby preventing phosphorylation of RB1 and blocking E2F-dependent transcription.
110	***EPB42***	15:43489425-43513323	yes	177070	Probably plays an important role in the regulation of erythrocyte shape and mechanical properties.
111	***TGM5***	15:43524793-43559055	yes	603805	Catalyzes the cross-linking of proteins and the conjugation of polyamines to proteins. Contributes to the formation of the cornified cell envelope of keratinocytes.
112	***ATP5HP1***	15:43560029-43560623	no	-	-
113	***TGM7***	15:43568479-43594453	yes	606776	Catalyzes the cross-linking of proteins and the conjugation of polyamines to proteins.
114	***LCMT2***	15:43619974-43622820	yes	611246	Probable S-adenosyl-L-methionine-dependent methyltransferase that acts as a component of the wybutosine biosynthesis pathway. Wybutosine is a hyper modified guanosine with a tricyclic base found at the 3'-position adjacent to the anticodon of eukaryotic phenylalanine tRNA (By similarity). May methylate the carboxyl group of leucine residues to form alpha-leucine ester residues.

115	***ADAL***	15:43622549-43646753	yes	619346	Catalyzes the hydrolysis of the free cytosolic methylated adenosine nucleotide N(6)-methyl-AMP (N6-mAMP) to produce inositol monophosphate (IMP) and methylamine (PubMed:21755941, PubMed:29884623). Is required for the catabolism of cytosolic N6-mAMP, which is derived from the degradation of mRNA containing N6-methylated adenine (m6A) (PubMed:21755941, PubMed:29884623). Catalyzes the removal of different alkyl groups not only from N6-substituted purine or 2-aminopurine nucleoside monophosphates but also from O6-substituted compounds in vitro (PubMed:21755941).
116	***ZSCAN29***	15:43650370-43662258	yes	-	May be involved in transcriptional regulation.
117	***TUBGCP4***	15:43663257-43699293	yes	609610	Gamma-tubulin complex is necessary for microtubule nucleation at the centrosome.
118	***RN7SL487P***	15:43683582-43683925	no	-	-
119	***TP53BP1***	15:43699412-43802707	yes	605230	Double-strand break (DSB) repair protein involved in response to DNA damage, telomere dynamics and class-switch recombination (CSR) during antibody genesis (PubMed:12364621, PubMed:22553214, PubMed:23333306, PubMed:17190600, PubMed:21144835, PubMed:27153538, PubMed:28241136). Plays a key role in the repair of double-strand DNA breaks

					(DSBs) in response to DNA damage by promoting non-homologous end joining (NHEJ)-mediated repair of DSBs and specifically counteracting the function of the homologous recombination (HR) repair protein BRCA1 (PubMed:22553214, PubMed:23727112, PubMed:23333306, PubMed:27153538). In response to DSBs, phosphorylation by ATM promotes interaction with RIF1 and dissociation from NUDT16L1/TIRR, leading to recruitment to DSBs sites (PubMed:28241136). Recruited to DSBs sites by recognizing and binding histone H2A monoubiquitinated at 'Lys-15' (H2AK15Ub) and histone H4 dimethylated at 'Lys-20' (H4K20me2), two histone marks that are present at DSBs sites (PubMed:23760478, PubMed:27153538, PubMed:28241136, PubMed:17190600). Required for immunoglobulin class-switch recombination (CSR) during antibody genesis, a process that involves the generation of DNA DSBs (PubMed:23345425). Participates in the repair and the orientation of the broken DNA ends during CSR (By similarity). In contrast, it is not required for classic NHEJ and V(D)J recombination (By similarity). Promotes NHEJ of dysfunctional telomeres via interaction with PAXIP1

					(PubMed:23727112).
120	***MAP1A***	15:43809806-43823818	yes	600178	Structural protein involved in the filamentous cross-bridging between microtubules and other skeletal elements.
121	***PPIP5K1***	15:43825660-43882451	yes	610979	Bifunctional inositol kinase that acts in concert with the IP6K kinases IP6K1, IP6K2 and IP6K3 to synthesize the diphosphate group-containing inositol pyrophosphates diphosphoinositol pentakisphosphate, PP-InsP5, and bis-diphosphoinositol tetrakisphosphate, (PP)2-InsP4. PP-InsP5 and (PP)2-InsP4, also respectively called InsP7 and InsP8, regulate a variety of cellular processes, including apoptosis, vesicle trafficking, cytoskeletal dynamics, exocytosis, insulin signaling and neutrophil activation. Phosphorylates inositol hexakisphosphate (InsP6) at position 1 to produce PP-InsP5 which is in turn phosphorylated by IP6Ks to produce (PP)2-InsP4. Alternatively, phosphorylates PP-InsP5 at position 1, produced by IP6Ks from InsP6, to produce (PP)2-InsP4. Activated when cells are exposed to hyperosmotic stress.
122	***CKMT1B***	15:43885055-43891604	yes	123290	Reversibly catalyzes the transfer of phosphate between ATP and various phosphogens (e.g. creatine phosphate). Creatine kinase isoenzymes play a central role in energy transduction in tissues

					with large, fluctuating energy demands, such as skeletal muscle, heart, brain and spermatozoa.
123	***STRC***	15:43891761-43910998	yes	606440	Essential to the formation of horizontal top connectors between outer hair cell stereocilia.
124	***RNU6-554P***	15:43894741-43894846	no	-	-
125	***CATSPER2***	15:43922760-43941043	yes	607249	Voltage-gated calcium channel that plays a central role in calcium-dependent physiological responses essential for successful fertilization, such as sperm hyperactivation, acrosome reaction and chemotaxis towards the oocyte.
126	***RNU6-610P***	15:43929830-43929936	no	-	-
127	***PDIA3P2***	15:43941174-43941489	no	-	-
128	***PPIP5K1P1***	15:43956615-43976925	no	-	-
129	***CKMT1A***	15:43984984-43991420	yes	613415	Reversibly catalyzes the transfer of phosphate between ATP and various phosphogens (e.g. creatine phosphate). Creatine kinase isoenzymes play a central role in energy transduction in tissues with large, fluctuating energy demands, such as skeletal muscle, heart, brain and spermatozoa.
130	***STRCP1***	15:43991577-44010460	no	-	-
131	***RNU6-353P***	15:43994561-43994668	no	-	-
132	***RNU6-354P***	15:44026377-44026483	no	-	-
133	***CATSPER2P1***	15:44028132-44038496	no	-	-
134	***PDIA3***	15:44038590-44064804	yes	602046	Disulfide isomerase which catalyzes the formation, isomerization, and reduction or oxidation of disulfide bonds (PubMed:7487104,

					PubMed:27897272). Associates with calcitriol, the active form of vitamin D3 which mediates the action of this vitamin on cells (PubMed:27897272). Association with calcitriol does not affect its enzymatic activity (PubMed:27897272).
135	***ELL3***	15:44064798-44069502	yes	609885	Enhancer-binding elongation factor that specifically binds enhancers in embryonic stem cells (ES cells), marks them, and is required for their future activation during stem cell specification. Does not only bind to enhancer regions of active genes, but also marks the enhancers that are in a poised or inactive state in ES cells and is required for establishing proper RNA polymerase II occupancy at developmentally regulated genes in a cohesin-dependent manner. Probably required for priming developmentally regulated genes for later recruitment of the super elongation complex (SEC), for transcriptional activation during differentiation. Required for recruitment of P-TEFb within SEC during differentiation. Probably preloaded on germ cell chromatin, suggesting that it may prime gene activation by marking enhancers as early as in the germ cells. Promoting epithelial-mesenchymal transition (EMT) (By similarity). Elongation factor component of the super elongation complex (SEC), a complex required to increase the catalytic rate of

					RNA polymerase II transcription by suppressing transient pausing by the polymerase at multiple sites along the DNA. Component of the little elongation complex (LEC), a complex required to regulate small nuclear RNA (snRNA) gene transcription by RNA polymerase II and III (PubMed:22195968).
136	***SERF2***	15:44069294-44088287	yes	605054	Positive regulator of amyloid protein aggregation and proteotoxicity (PubMed:20723760). Induces conformational changes in amyloid proteins, such as HTT, driving them into compact formations preceding the formation of aggregates (PubMed:20723760).
137	***SERF2-C15ORF63***	15:44084174-44094787	no	-	-
138	***MIR1282***	15:44085857-44085957	no	-	-
139	***SERINC4***	15:44086372-44092295	yes	614550	Incorporates a polar amino acid serine into membranes and facilitates the synthesis of two serine-derived lipids, phosphatidylserine and sphingolipids.
140	***HYPK***	15:44092619-44094787	yes	612784	Component of several N-terminal acetyltransferase complexes (PubMed:20154145, PubMed:29754825, PubMed:32042062). Inhibits the N-terminal acetylation activity of the N-terminal acetyltransferase NAA10-NAA15 complex (also called the NatA complex) (PubMed:29754825, PubMed:32042062). Has chaperone-like activity preventing polyglutamine (polyQ) aggregation of

					HTT in neuronal cells probably while associated with the NatA complex (PubMed:17947297, PubMed:20154145). May play a role in the NatA complex-mediated N-terminal acetylation of PCNP (PubMed:20154145).
141	***MFAP1***	15:44096733-44116951	yes	600215	Involved in pre-mRNA splicing as a component of the spliceosome.
142	***WDR76***	15:44119112-44160617	yes	-	Specifically binds 5-hydroxymethylcytosine (5hmC), suggesting that it acts as a specific reader of 5hmC.
143	***FRMD5***	15:44162959-44487492	yes	616309	May be involved in regulation of cell migration (PubMed:22846708, PubMed:25448675). May regulate cell-matrix interactions via its interaction with ITGB5 and modifying ITGB5 cytoplasmic tail interactions such as with FERMT2 and TLN1. May regulate ROCK1 kinase activity possibly involved in regulation of actin stress fiber formation (PubMed:25448675).
144	***PIN4P1***	15:44168004-44170369	no	-	-
145	***ACTBP7***	15:44280676-44282463	no	-	-
146	***GAPDHP55***	15:44355642-44356507	no	-	-
147	***GOLM2***	15:44580909-44707959	yes	-	The increased expression level of this gene is associated with HER-2/neu proto-oncogene overexpression. Amplification and resulting overexpression of this proto-oncogene are found in approximately 30% of human breast and 20% of

					human ovarian cancers. Alternatively spliced variants encoding different isoforms have been identified for this gene. [provided by RefSeq, Dec 2010]
148	***GAPDHP43***	15:44646806-44648100	no	-	-
149	***CTDSPL2***	15:44719579-44819430	yes	618739	Probable phosphatase.
150	***HNRNPMP1***	15:44739773-44741547	no	-	-
151	***EIF3J-DT***	15:44826703-44829121	no	-	-
152	***EIF3J***	15:44829266-44855001	yes	603910	Component of the eukaryotic translation initiation factor 3 (eIF-3) complex, which is required for several steps in the initiation of protein synthesis (PubMed:25849773, PubMed:27462815). The eIF-3 complex associates with the 40S ribosome and facilitates the recruitment of eIF-1, eIF-1A, eIF-2:GTP:methionyl-tRNAi and eIF-5 to form the 43S pre-initiation complex (43S PIC). The eIF-3 complex stimulates mRNA recruitment to the 43S PIC and scanning of the mRNA for AUG recognition. The eIF-3 complex is also required for disassembly and recycling of post-termination ribosomal complexes and subsequently prevents premature joining of the 40S and 60S ribosomal subunits prior to initiation. The eIF-3 complex specifically targets and initiates translation of a subset of mRNAs involved in cell proliferation, including cell cycling, differentiation and apoptosis,

					and uses different modes of RNA stem-loop binding to exert either translational activation or repression (PubMed:25849773).
153	***SPG11***	15:44854894-44955876	yes	610844	May play a role in neurite plasticity by maintaining cytoskeleton stability and regulating synaptic vesicle transport.
154	***PATL2***	15:44957930-44969086	yes	614661	RNA-binding protein that acts as a translational repressor.
155	***B2M***	15:45003685-45010357	yes	109700	Component of the class I major histocompatibility complex (MHC). Involved in the presentation of peptide antigens to the immune system. Exogenously applied M.tuberculosis EsxA or EsxA-EsxB (or EsxA expressed in host) binds B2M and decreases its export to the cell surface (total protein levels do not change), probably leading to defects in class I antigen presentation (PubMed:25356553).
156	***LOC100419583***	15:45021186-45025049	no	-	-
157	***TRIM69***	15:45028560-45060027	yes	616017	May have E3 ubiquitin-protein ligase activity. May play a role in apoptosis.
158	***SORD2P***	15:45117697-45139761	no	-	-
159	***RNU1-119P***	15:45176814-45176978	no	-	-
160	***LOC100420928***	15:45206818-45222820	no	-	-
161	***LOC100129830***	15:45212155-45213235	no	-	-
162	***RNU6-1108P***	15:45219803-45219901	no	-	-
163	***TERB2***	15:45248900-45271421	yes	617131	Meiosis-specific telomere-associated protein

					involved in meiotic telomere attachment to the nucleus inner membrane, a crucial step for homologous pairing and synapsis. Component of the MAJIN-TERB1-TERB2 complex, which promotes telomere cap exchange by mediating attachment of telomeric DNA to the inner nuclear membrane and replacement of the protective cap of telomeric chromosomes: in early meiosis, the MAJIN-TERB1-TERB2 complex associates with telomeric DNA and the shelterin/telosome complex. During prophase, the complex matures and promotes release of the shelterin/telosome complex from telomeric DNA.
164	***RNU6-1332P***	15:45255028-45255131	no	-	-
165	***LOC100187725***	15:45262012-45263512	no	-	-
166	***RNU6-966P***	15:45265756-45265858	no	-	-
167	***RNU1-78P***	15:45295062-45295226	no	-	-
168	***SORD***	15:45315302-45367287	yes	182500	Polyol dehydrogenase that catalyzes the reversible NAD(+)-dependent oxidation of various sugar alcohols. Is mostly active with D-sorbitol (D-glucitol), L-threitol, xylitol and ribitol as substrates, leading to the C2-oxidized products D-fructose, L-erythrulose, D-xylulose, and D-ribulose, respectively (PubMed:3365415). Is a key enzyme in the polyol pathway that interconverts glucose and fructose via sorbitol, which constitutes an important

					alternate route for glucose metabolism. The polyol pathway is believed to be involved in the etiology of diabetic complications, such as diabetic neuropathy and retinopathy, induced by hyperglycemia (PubMed:12962626, PubMed:29966615, PubMed:25105142). May play a role in sperm motility by using sorbitol as an alternative energy source for sperm motility (PubMed:16278369). May have a more general function in the metabolism of secondary alcohols since it also catalyzes the stereospecific oxidation of (2R,3R)-2,3-butanediol. To a lesser extent, can also oxidize L-arabinitol, galactitol and D-mannitol and glycerol in vitro. Oxidizes neither ethanol nor other primary alcohols. Cannot use NADP(+) as the electron acceptor (PubMed:3365415).
169	***LOC100422669***	15:45344876-45345855	no	-	-
170	***DUOX2***	15:45384852-45406359	yes	606759	Generates hydrogen peroxide which is required for the activity of thyroid peroxidase/TPO and lactoperoxidase/LPO. Plays a role in thyroid hormones synthesis and lactoperoxidase-mediated antimicrobial defense at the surface of mucosa. May have its own peroxidase activity through its N-terminal peroxidase-like domain.
171	***DUOXA2***	15:45406523-45410304	yes	612772	Required for the maturation and the transport from the endoplasmic reticulum to the plasma membrane

					of functional DUOX2. May play a role in thyroid hormone synthesis.
172	***DUOXA1***	15:45409564-45422075	yes	612771	May be required for the maturation and the transport from the endoplasmic reticulum to the plasma membrane of functional DUOX1.
173	***DUOX1***	15:45422192-45457776	yes	606758	Generates hydrogen peroxide which is required for the activity of thyroid peroxidase/TPO and lactoperoxidase/LPO. Plays a role in thyroid hormones synthesis and lactoperoxidase-mediated antimicrobial defense at the surface of mucosa. May have its own peroxidase activity through its N-terminal peroxidase-like domain.
174	***SHF***	15:45459412-45493373	yes	617313	Adapter protein which may play a role in the regulation of apoptosis in response to PDGF.
175	***TRH-GTG1-7***	15:45490804-45490875	no	-	-
176	***TRH-GTG1-8***	15:45492611-45492682	no	-	-
177	***TRH-GTG1-9***	15:45493349-45493420	no	-	-
178	***LOC101928414***	15:45543779-45571420	no	-	-
179	***SLC28A2***	15:45544428-45568132	yes	606208	Sodium-dependent and purine-selective transporter (PubMed:9435697, PubMed:10087507). Exhibits the transport characteristics of the nucleoside transport system cif or N1 subtype (N1/cif) (selective for purine nucleosides and uridine) (PubMed:9435697, PubMed:10087507). Plays a critical role in specific uptake and salvage of purine nucleosides in kidney and other tissues

					(PubMed:9435697).
180	***LOC100533853***	15:45624005-45624822	no	-	-
181	***RNU6-953P***	15:45646978-45647083	no	-	-
182	***GATM***	15:45653322-45694515	yes	602360	Catalyzes the biosynthesis of guanidinoacetate, the immediate precursor of creatine. Creatine plays a vital role in energy metabolism in muscle tissues. May play a role in embryonic and central nervous system development. May be involved in the response to heart failure by elevating local creatine synthesis.
183	***LOC145663***	15:45670898-45672321	no	-	-
184	***SPATA5L1***	15:45694519-45713617	yes	619578	ATP-dependent chaperone, which plays an essential role in the cytoplasmic maturation steps of pre-60S ribosomal particles by promoting the release of shuttling protein RSL24D1/RLP24 from the pre-ribosomal particles (PubMed:35354024). Acts together with SPATA5, C1orf109 and CINP (PubMed:35354024).
185	***C15orf48***	15:45722727-45725647	yes	608409	This gene was first identified in a study of human esophageal squamous cell carcinoma tissues. Levels of both the message and protein are reduced in carcinoma samples. In adult human tissues, this gene is expressed in the the esophagus, stomach, small intestine, colon and placenta. Alternatively spliced transcript variants that encode the same protein have been identified. [provided by RefSeq,

					Jun 2012]
186	***MIR147B***	15:45725248-45725327	no	-	-
187	***SLC30A4***	15:45771811-45815002	yes	602075	Zinc ion transporter mediating zinc import from cytoplasm potentially into the endocytic compartment (PubMed:19521526). Controls zinc deposition in milk (By similarity).
188	***HMGN2P46***	15:45803334-45848928	no	611314	-
189	***DPPA5P2***	15:45858972-45859241	no	-	-
190	***BLOC1S6***	15:45879321-45901914	yes	604310	Component of the BLOC-1 complex, a complex that is required for normal biogenesis of lysosome-related organelles (LRO), such as platelet dense granules and melanosomes. In concert with the AP-3 complex, the BLOC-1 complex is required to target membrane protein cargos into vesicles assembled at cell bodies for delivery into neurites and nerve terminals. The BLOC-1 complex, in association with SNARE proteins, is also proposed to be involved in neurite extension. May play a role in intracellular vesicle trafficking, particularly in the vesicle-docking and fusion process.
191	***SQOR***	15:45923346-45983492	yes	617658	Catalyzes the oxidation of hydrogen sulfide with the help of a quinone, such as ubiquinone-10, giving rise to thiosulfate and ultimately to sulfane (molecular sulfur) atoms. Requires an additional electron acceptor; can use sulfite, sulfide or cyanide (in vitro) (PubMed:22852582). It is believed the in

					vivo electron acceptor is glutathione (PubMed:25225291,PubMed:29715001).
192	***LOC105370802***	15:45997393-46223267	no	-	-
193	***LOC100419366***	15:46391401-46392572	no	-	-
194	***RNU6-1014P***	15:46952856-46952958	no	-	-
195	***RN7SKP101***	15:47012391-47012710	no	-	-
196	***LOC729316***	15:47029645-47039377	no	-	-
197	***SEMA6D***	15:47476403-48066420	yes	609295	Shows growth cone collapsing activity on dorsal root ganglion (DRG) neurons in vitro. May be a stop signal for the DRG neurons in their target areas, and possibly also for other neurons. May also be involved in the maintenance and remodeling of neuronal connections.
198	***RN7SKP139***	15:47564795-47565026	no	-	-
199	***RPL7AP62***	15:48022280-48023157	no	-	-
200	***LINC01491***	15:48095581-48138433	no	-	-
201	***SLC24A5***	15:48413169-48434589	yes	609802	Calcium, potassium:sodium antiporter that transports 1 Ca(2+) and 1 K(+) to the melanosome in exchange for 4 cytoplasmic Na(+) (PubMed:18166528). Involved in pigmentation, possibly by participating in ion transport in melanosomes (PubMed:16357253, PubMed:18166528). Predominant sodium-calcium exchanger in melanocytes (PubMed:16357253, PubMed:18166528).
202	***MYEF2***	15:48431625-48470558	yes	619395	Transcriptional repressor of the myelin basic

					protein gene (MBP). Binds to the proximal MB1 element 5'-TTGTCC-3' of the MBP promoter. Its binding to MB1 and function are inhibited by PURA (By similarity).
203	***CTXN2***	15:48483867-48495951	yes	-	Predicted to be integral component of membrane. [provided by Alliance of Genome Resources, Apr 2022]
204	***SLC12A1***	15:48498498-48596275	yes	600839	Na+/K+/Cl- Symporters (NKCC) are membrane-bound channels that play a major role in a variety of epithelial absorptive and secretory processes and a direct role in cell volume regulation and possibly in cell cycle regulation. There are two NKCC isoforms: NKCC1 and NKCC2.
205	***DUT***	15:48623292-48635570	yes	601266	Catalyzes the cleavage of 2'-deoxyuridine 5'-triphosphate (dUTP) into 2'-deoxyuridine 5'-monophosphate (dUMP) and inorganic pyrophosphate and through its action efficiently prevents uracil misincorporation into DNA and at the same time provides dUMP, the substrate for de novo thymidylate biosynthesis (PubMed:17880943, PubMed:8631816, PubMed:8805593). Inhibits peroxisome proliferator-activated receptor (PPAR) activity by binding of its N-terminal to PPAR, preventing the latter's dimerization with retinoid X receptor (By similarity). Essential for embryonic development (By similarity).

206	***FBN1***	15:48700503-48937985	yes	134797	[Asprosin]: Adipokine secreted by white adipose tissue that plays an important regulatory role in the glucose metabolism of liver, muscle and pancreas (PubMed:27087445, PubMed:30853600). Hormone that targets the liver in response to fasting to increase plasma glucose levels (PubMed:27087445). Binds the olfactory receptor OR4M1 at the surface of hepatocytes and promotes hepatocyte glucose release by activating the protein kinase A activity in the liver, resulting in rapid glucose release into the circulation (PubMed:27087445, PubMed:31230984). May act as a regulator of adaptive thermogenesis by inhibiting browning and energy consumption, while increasing lipid deposition in white adipose tissue (By similarity). Also acts as an orexigenic hormone that increases appetite: crosses the blood brain barrier and exerts effects on the hypothalamus (By similarity). In the arcuate nucleus of the hypothalamus, asprosin directly activates orexigenic AgRP neurons and indirectly inhibits anorexigenic POMC neurons, resulting in appetite stimulation (By similarity). Activates orexigenic AgRP neurons via binding to the olfactory receptor OR4M1 (By similarity). May also play a role in sperm motility in testis via interaction with OR4M1

					receptor (By similarity).||[Fibrillin-1]: Structural component of the 10-12 nm diameter microfibrils of the extracellular matrix, which conveys both structural and regulatory properties to load-bearing connective tissues (PubMed:1860873, PubMed:15062093). Fibrillin-1-containing microfibrils provide long-term force bearing structural support (PubMed:27026396). In tissues such as the lung, blood vessels and skin, microfibrils form the periphery of the elastic fiber, acting as a scaffold for the deposition of elastin (PubMed:27026396). In addition, microfibrils can occur as elastin-independent networks in tissues such as the ciliary zonule, tendon, cornea and glomerulus where they provide tensile strength and have anchoring roles (PubMed:27026396). Fibrillin-1 also plays a key role in tissue homeostasis through specific interactions with growth factors, such as the bone morphogenetic proteins (BMPs), growth and differentiation factors (GDFs) and latent transforming growth factor-beta-binding proteins (LTBPs), cell-surface integrins and other extracellular matrix protein and proteoglycan components (PubMed:27026396). Regulates osteoblast maturation by controlling TGF-beta bioavailability and calibrating TGF-beta and BMP

					levels, respectively (By similarity). Negatively regulates osteoclastogenesis by binding and sequestering an osteoclast differentiation and activation factor TNFSF11 (PubMed:24039232). This leads to disruption of TNFSF11-induced Ca(2+) signaling and impairment of TNFSF11-mediated nuclear translocation and activation of transcription factor NFATC1 which regulates genes important for osteoclast differentiation and function (PubMed:24039232). Mediates cell adhesion via its binding to cell surface receptors integrins ITGAV:ITGB3 and ITGA5:ITGB1 (PubMed:12807887, PubMed:17158881). Binds heparin and this interaction has an important role in the assembly of microfibrils (PubMed:11461921).
207	***LOC100506059***	15:48938854-48944213	no	-	-
208	***LOC645405***	15:49017542-49018025	no	-	-
209	***CEP152***	15:49030135-49103343	yes	613529	Necessary for centrosome duplication; the function seems also to involve CEP63, CDK5RAP2 and WDR62 through a stepwise assembled complex at the centrosome that recruits CDK2 required for centriole duplication (PubMed:26297806). Acts as a molecular scaffold facilitating the interaction of PLK4 and CENPJ, 2 molecules involved in centriole formation (PubMed:21059844, PubMed:20852615). Proposed to snatch PLK4

					away from PLK4:CEP92 complexes in early G1 daughter centriole and to reposition PLK4 at the outer boundary of a newly forming CEP152 ring structure (PubMed:24997597). Also plays a key role in deuterosome-mediated centriole amplification in multiciliated that can generate more than 100 centrioles (By similarity). Overexpression of CEP152 can drive amplification of centrioles (PubMed:20852615).
210	***LOC100506104***	15:49102895-49104106	no	-	-
211	***SHC4***	15:49115934-49255641	yes	617372	Activates both Ras-dependent and Ras-independent migratory pathways in melanomas. Contributes to the early phases of agrin-induced tyrosine phosphorylation of CHRNB1.
212	***EID1***	15:49170290-49172380	yes	605894	Interacts with RB1 and EP300 and acts as a repressor of MYOD1 transactivation. Inhibits EP300 and CBP histone acetyltransferase activity. May be involved in coupling cell cycle exit to the transcriptional activation of genes required for cellular differentiation. May act as a candidate coinhibitory factor for NR0B2 that can be directly linked to transcription inhibitory mechanisms.
213	***KRT8P24***	15:49263922-49265761	no	-	-
214	***SECISBP2L***	15:49280835-49338760	yes	615756	Binds SECIS (Sec insertion sequence) elements present on selenocysteine (Sec) protein mRNAs, but does not promote Sec incorporation into

					selenoproteins in vitro.
215	***RN7SL577P***	15:49334538-49334840	no	-	-
216	***COPS2***	15:49417471-49447854	yes	604508	Essential component of the COP9 signalosome complex (CSN), a complex involved in various cellular and developmental processes. The CSN complex is an essential regulator of the ubiquitin (Ubl) conjugation pathway by mediating the deneddylation of the cullin subunits of SCF-type E3 ligase complexes, leading to decrease the Ubl ligase activity of SCF-type complexes such as SCF, CSA or DDB2. The complex is also involved in phosphorylation of p53/TP53, c-jun/JUN, IkappaBalpha/NFKBIA, ITPK1 and IRF8/ICSBP, possibly via its association with CK2 and PKD kinases. CSN-dependent phosphorylation of TP53 and JUN promotes and protects degradation by the Ubl system, respectively. Involved in early stage of neuronal differentiation via its interaction with NIF3L1.
217	***GALK2***	15:49447956-49622002	yes	137028	Acts on GalNAc. Also acts as a galactokinase when galactose is present at high concentrations. May be involved in a salvage pathway for the reutilization of free GalNAc derived from the degradation of complex carbohydrates.
218	***NDUFAF4P1***	15:49448495-49450822	no	-	-
219	***MIR4716***	15:49461267-49461350	no	-	-

220	***RPL15P19***	15:49469498-49470992	no	-	-
221	***RN7SL307P***	15:49491097-49491391	no	-	-
222	***FAM227B***	15:49620590-49913128	yes	-	FAM227B (Family With Sequence Similarity 227 Member B) is a Protein Coding gene.
223	***LOC100420615***	15:49657062-49659024	no	-	-
224	***FGF7***	15:49715375-49779523	yes	148180	Plays an important role in the regulation of embryonic development, cell proliferation and cell differentiation. Required for normal branching morphogenesis. Growth factor active on keratinocytes. Possible major paracrine effector of normal epithelial cell proliferation.
225	***DTWD1***	15:49913226-49937333	yes	-	Catalyzes the formation of 3-(3-amino-3-carboxypropyl) uridine (acp3U) at position 20 in the D-loop of several cytoplasmic tRNAs (acp3U (20)).
226	***RLIMP3***	15:50086441-50088294	no	-	-
227	***ATP8B4***	15:50150435-50411419	yes	609123	Component of a P4-ATPase flippase complex which catalyzes the hydrolysis of ATP coupled to the transport of aminophospholipids from the outer to the inner leaflet of various membranes and ensures the maintenance of asymmetric distribution of phospholipids. Phospholipid translocation seems also to be implicated in vesicle formation and in uptake of lipid signaling molecules (Probable).
228	***RNA5SP394***	15:50274640-50274758	no	-	-
229	***SLC27A2***	15:50474393-50528589	yes	603247	[Isoform 1]: Exhibits both long-chain fatty acids (LCFA) transport activity and acyl CoA synthetase

					towards very long-chain fatty acids (PubMed:21768100, PubMed:10198260). Shows a preference for generating CoA derivatives of n-3 fatty acids, which are preferentially trafficked into phosphatidylinositol (PubMed:21768100).‖[Isoform 2]: Exhibits long-chain fatty acids (LCFA) transport activity but lacks acyl CoA synthetase towards very long-chain fatty acids.‖Mediates the import of long-chain fatty acids (LCFA) into the cell by facilitating their transport across cell membranes, playing an important role in hepatic fatty acid uptake (PubMed:20530735, PubMed:22022213, PubMed:24269233, PubMed:10198260, PubMed:10749848, PubMed:11980911). Also functions as an acyl-CoA ligase catalyzing the ATP-dependent formation of fatty acyl-CoA using LCFA and very-long-chain fatty acids (VLCFA) as substrates, which prevents fatty acid efflux from cells and might drive more fatty acid uptake (PubMed:20530735, PubMed:22022213, PubMed:24269233, PubMed:10198260, PubMed:10749848, PubMed:11980911). Plays a pivotal role in regulating available LCFA substrates from exogenous sources in tissues undergoing high levels of beta-oxidation or triglyceride synthesis

					(PubMed:20530735). Can also activate branched-chain fatty acids such as phytanic acid and pristanic acid (PubMed:10198260). May contribute to the synthesis of sphingosine-1-phosphate (PubMed:24269233). Does not activate C24 bile acids, cholate and chenodeoxycholate (PubMed:11980911). In vitro, activates 3-alpha,7-alpha,12-alpha-trihydroxy-5-beta-cholestanate (THCA), the C27 precursor of cholic acid deriving from the de novo synthesis from cholesterol (PubMed:11980911). However, it is not critical for THCA activation and bile synthesis in vivo (PubMed:20530735).
230	***HDC***	15:50534144-50558162	yes	142704	Catalyzes the biosynthesis of histamine from histidine.
231	***RN7SL494P***	15:50536825-50537128	no	-	-
232	***GABPB1***	15:50569389-50647626	yes	600610	(Microbial infection) Necessary for the expression of the Adenovirus E4 gene.||Transcription factor capable of interacting with purine rich repeats (GA repeats) (PubMed:8441384, PubMed:10675337, PubMed:8816484). Acts as a a master regulator of nuclear-encoded mitochondrial genes (By similarity).
233	***RNU6-94P***	15:50639622-50639726	no	-	-
234	***GABPB1-IT1***	15:50641133-50647076	no	-	-
235	***GABPB1-AS1***	15:50646371-50650509	no	-	-

236	***MIR4712***	15:50652526-50652607	no	-	-
237	***AHCYP7***	15:50709486-50711080	no	-	-
238	***USP8***	15:50716574-50793280	yes	603158	Hydrolase that can remove conjugated ubiquitin from proteins and therefore plays an important regulatory role at the level of protein turnover by preventing degradation. Converts both 'Lys-48' an 'Lys-63'-linked ubiquitin chains. Catalytic activity is enhanced in the M phase. Involved in cell proliferation. Required to enter into S phase in response to serum stimulation. May regulate T-cell anergy mediated by RNF128 via the formation of a complex containing RNF128 and OTUB1. Probably regulates the stability of STAM2 and RASGRF1. Regulates endosomal ubiquitin dynamics, cargo sorting, membrane traffic at early endosomes, and maintenance of ESCRT-0 stability. The level of protein ubiquitination on endosomes is essential for maintaining the morphology of the organelle. Deubiquitinates EPS15 and controles tyrosine kinase stability. Removes conjugated ubiquitin from EGFR thus regulating EGFR degradation and downstream MAPK signaling. Involved in acrosome biogenesis through interaction with the spermatid ESCRT-0 complex and microtubules. Deubiquitinates BIRC6/bruce and KIF23/MKLP1. Deubiquitinates BACE1 which

					inhibits BACE1 lysosomal degradation and modulates BACE-mediated APP cleavage and amyloid-beta formation (PubMed:27302062).
239	***RNA5SP395***	15:50755578-50755695	no	-	-
240	***RPS20P34***	15:50758817-50759335	no	-	-
241	***USP50***	15:50792759-50838902	yes	-	Has no peptidase activity.
242	***TRPM7***	15:50849355-50979012	yes	605692	Essential ion channel and serine/threonine-protein kinase. Divalent cation channel permeable to calcium and magnesium (PubMed:35561741). Has a central role in magnesium ion homeostasis and in the regulation of anoxic neuronal cell death. Involved in TNF-induced necroptosis downstream of MLKL by mediating calcium influx. The kinase activity is essential for the channel function. May be involved in a fundamental process that adjusts plasma membrane divalent cation fluxes according to the metabolic state of the cell. Phosphorylates annexin A1 (ANXA1).
243	***RN7SL354P***	15:50981097-50981312	no	-	-
244	***SPPL2A***	15:50999730-51057910	yes	608238	Intramembrane-cleaving aspartic protease (I-CLiP) that cleaves type II membrane signal peptides in the hydrophobic plane of the membrane. Functions in FASLG, ITM2B and TNF processing (PubMed:16829952, PubMed:16829951, PubMed:17557115, PubMed:17965014). Catalyzes the intramembrane cleavage of the anchored

					fragment of shed TNF-alpha (TNF), which promotes the release of the intracellular domain (ICD) for signaling to the nucleus (PubMed:16829952). Also responsible for the intramembrane cleavage of Fas antigen ligand FASLG, which promotes the release of the intracellular FasL domain (FasL ICD) (PubMed:17557115). Essential for degradation of the invariant chain CD74 that plays a central role in the function of antigen-presenting cells in the immune system (By similarity). Plays a role in the regulation of innate and adaptive immunity (PubMed:16829952). Catalyzes the intramembrane cleavage of the simian foamy virus envelope glycoprotein gp130 independently of prior ectodomain shedding by furin or furin-like proprotein convertase (PC)-mediated cleavage proteolysis (PubMed:23132852).
245	***RPL32P30***	15:51090867-51091154	no	-	-
246	***AP4E1***	15:51200869-51298097	yes	607244	Component of the adaptor protein complex 4 (AP-4). Adaptor protein complexes are vesicle coat components involved both in vesicle formation and cargo selection. They control the vesicular transport of proteins in different trafficking pathways (PubMed:10066790, PubMed:10436028). AP-4 forms a non clathrin-associated coat on vesicles

					departing the trans-Golgi network (TGN) and may be involved in the targeting of proteins from the trans-Golgi network (TGN) to the endosomal-lysosomal system. It is also involved in protein sorting to the basolateral membrane in epithelial cells and the proper asymmetric localization of somatodendritic proteins in neurons. AP-4 is involved in the recognition and binding of tyrosine-based sorting signals found in the cytoplasmic part of cargos, but may also recognize other types of sorting signal (Probable).
247	***DCAF13P3***	15:51236326-51238770	no	-	-
248	***TNFAIP8L3***	15:51348798-51397473	yes	616483	Acts as a lipid transfer protein. Preferentially captures and shuttles two lipid second messengers, i.e., phosphatidylinositol 4,5- bisphosphate and phosphatidylinositol 3,4,5-trisphosphate and increases their levels in the plasma membrane. Additionally, may also function as a lipid-presenting protein to enhance the activity of the PI3K-AKT and MEK-ERK pathways. May act as a regulator of tumorigenesis through its activation of phospholipid signaling.
249	***CYP19A1***	15:51500254-51630795	yes	107910	Cytochrome P450 (CYP450) enzymes are a diverse group of catalysts that contains 57 members in humans. CYPs are usually membrane-bound and are localized to the inner mitochondrial or

					endoplasmic reticular membrane. CYPs have oxygenase activity.
250	***MIR4713***	15:51534387-51534461	no	-	-
251	***MIR7973-2***	15:51606229-51606304	no	-	-
252	***MIR7973-1***	15:51606231-51606306	no	-	-
253	***LOC100506192***	15:51629124-51632214	no	-	-
254	***GLDN***	15:51633713-51700210	yes	608603	Ligand for NRCAM and NFASC/neurofascin that plays a role in the formation and maintenance of the nodes of Ranvier on myelinated axons. Mediates interaction between Schwann cell microvilli and axons via its interactions with NRCAM and NFASC. Nodes of Ranvier contain clustered sodium channels that are crucial for the saltatory propagation of action potentials along myelinated axons. During development, nodes of Ranvier are formed by the fusion of two heminodes. Required for normal clustering of sodium channels at heminodes; not required for the formation of mature nodes with normal sodium channel clusters. Required, together with NRCAM, for maintaining NFASC and sodium channel clusters at mature nodes of Ranvier.
255	***DMXL2***	15:51739921-51914967	yes	612186	May serve as a scaffold protein for MADD and RAB3GA on synaptic vesicles (PubMed:11809763). Plays a role in the brain as a key controller of neuronal and endocrine

					homeostatic processes (By similarity).
256	***SCG3***	15:51973550-52013223	yes	611796	Member of the granin protein family that regulates the biogenesis of secretory granules (PubMed:19357184). Acts as a sorting receptor for intragranular proteins including chromogranin A/CHGA (By similarity). May also play a role in angiogenesis. Promotes endothelial proliferation, migration and tube formation through MEK/ERK signalling pathway (PubMed:29154827).
257	***LYSMD2***	15:52015261-52043650	yes	-	LYSMD2 (LysM Domain Containing 2) is a Protein Coding gene. An important paralog of this gene is LYSMD1.LYSMD1 (LysM Domain Containing 1) is a Protein Coding gene. Diseases associated with LYSMD1 include 3-Methylglutaconic Aciduria with Cataracts, Neurologic Involvement And Neutropenia. An important paralog of this gene is LYSMD2.
258	***TMOD2***	15:52043758-52108560	yes	602928	Blocks the elongation and depolymerization of the actin filaments at the pointed end. The Tmod/TM complex contributes to the formation of the short actin protofilament, which in turn defines the geometry of the membrane skeleton (By similarity).
259	***TMOD3***	15:52121825-52204335	yes	605112	Blocks the elongation and depolymerization of the actin filaments at the pointed end. The Tmod/TM complex contributes to the formation of the short actin protofilament, which in turn defines the

					geometry of the membrane skeleton (By similarity).
260	***LOC100422490***	15:52151191-52153551	no	-	-
261	***RNU6-90P***	15:52199397-52199502	no	-	-
262	***LOC100422556***	15:52211160-52216317	no	-	-
263	***LEO1***	15:52230222-52263998	yes	610507	Component of the PAF1 complex (PAF1C) which has multiple functions during transcription by RNA polymerase II and is implicated in regulation of development and maintenance of embryonic stem cell pluripotency. PAF1C associates with RNA polymerase II through interaction with POLR2A CTD non-phosphorylated and 'Ser-2'- and 'Ser-5'-phosphorylated forms and is involved in transcriptional elongation, acting both independently and synergistically with TCEA1 and in cooperation with the DSIF complex and HTATSF1. PAF1C is required for transcription of Hox and Wnt target genes. PAF1C is involved in hematopoiesis and stimulates transcriptional activity of KMT2A/MLL1; it promotes leukemogenesis through association with KMT2A/MLL1-rearranged oncoproteins, such as KMT2A/MLL1-MLLT3/AF9 and KMT2A/MLL1-MLLT1/ENL. PAF1C is involved in histone modifications such as ubiquitination of histone H2B and methylation on histone H3 'Lys-4' (H3K4me3). PAF1C recruits the RNF20/40 E3 ubiquitin-protein

					ligase complex and the E2 enzyme UBE2A or UBE2B to chromatin which mediate mono-ubiquitination of 'Lys-120' of histone H2B (H2BK120ub1); UB2A/B-mediated H2B ubiquitination is proposed to be coupled to transcription. PAF1C is involved in mRNA 3' end formation probably through association with cleavage and poly(A) factors. In case of infection by influenza A strain H3N2, PAF1C associates with viral NS1 protein, thereby regulating gene transcription. Involved in polyadenylation of mRNA precursors. Connects PAF1C to Wnt signalling.
264	***MAPK6***	15:52311411-52358462	yes	602904	Atypical MAPK protein. Phosphorylates microtubule-associated protein 2 (MAP2) and MAPKAPK5. The precise role of the complex formed with MAPKAPK5 is still unclear, but the complex follows a complex set of phosphorylation events: upon interaction with atypical MAPKAPK5, ERK3/MAPK6 is phosphorylated at Ser-189 and then mediates phosphorylation and activation of MAPKAPK5, which in turn phosphorylates ERK3/MAPK6. May promote entry in the cell cycle (By similarity).
265	***RPS13P8***	15:52387469-52387986	no	-	-
266	***BCL2L10***	15:52401460-52404972	yes	606910	Promotes cell survival by suppressing apoptosis

					induced by BAX but not BAK (PubMed:11689480, PubMed:11278245). Increases binding of AHCYL1/IRBIT to ITPR1 (PubMed:27995898). Reduces ITPR1-mediated calcium release from the endoplasmic reticulum cooperatively with AHCYL1/IRBIT under normal cellular conditions (PubMed:27995898). Under apoptotic stress conditions, dissociates from ITPR1 and is displaced from mitochondria-associated endoplasmic reticulum membranes, leading to increased Ca (2+) transfer to mitochondria which promotes apoptosis (PubMed:27995898). Required for the correct formation of the microtubule organizing center during oocyte cell division, potentially via regulation of protein abundance and localization of other microtubule organizing center components such as AURKA and TPX2 (By similarity).
267	***GNB5***	15:52413123-52483565	yes	604447	Heterotrimeric G proteins are membrane bound GTPases that are linked to 7-TM receptors. Each G protein contains an alpha-, beta- and gamma-subunit and is bound to GDP in the 'off' state. Ligand binding causes a receptor conformational change, detaching the G protein and switching it 'on'.
268	***MYO5C***	15:52484515-52587995	yes	610022	May be involved in transferrin trafficking. Likely to power actin-based membrane trafficking in many

					physiologically crucial tissues.
269	***MIR1266***	15:52569314-52569397	no	-	-
270	***MYO5A***	15:52599480-52821247	yes	160777	Processive actin-based motor that can move in large steps approximating the 36-nm pseudo-repeat of the actin filament. Involved in melanosome transport. Also mediates the transport of vesicles to the plasma membrane. May also be required for some polarization process involved in dendrite formation.
271	***EEF1B2P1***	15:52797226-52798105	no	-	-
272	***ARPP19***	15:52839242-52861643	yes	605487	Protein phosphatase inhibitor that specifically inhibits protein phosphatase 2A (PP2A) during mitosis. When phosphorylated at Ser-62 during mitosis, specifically interacts with PPP2R2D (PR55-delta) and inhibits its activity, leading to inactivation of PP2A, an essential condition to keep cyclin-B1-CDK1 activity high during M phase. May indirectly enhance GAP-43 expression.
273	***FAM214A***	15:52873518-52970831	yes	-	ATOSA (Atos Homolog A) is a Protein Coding gene. An important paralog of this gene is ATOSB.ATOSA (Atos Homolog A) is a Protein Coding gene. An important paralog of this gene is ATOSB.
274	***ONECUT1***	15:53049160-53082209	yes	604164	Transcriptional activator. Binds the consensus sequence 5'-DHWATTGAYTWWD-3' on a variety of gene promoters such as those of HNF3B and TTR. Important for liver genes transcription.

275	***RPSAP55***	15:53177716-53178739	no	-	-
276	***EEF1A1P22***	15:53229336-53230715	no	-	-
277	***WDR72***	15:53805938-54055075	yes	613214	Plays a major role in formation of tooth enamel (PubMed:19853237, PubMed:25008349). Specifically required during the maturation phase of amelogenesis for normal formation of the enamel matrix and clearance of enamel proteins. May be involved in localization of the calcium transporter SLC24A4 to the ameloblast cell membrane.
278	***RNU2-53P***	15:53944174-53944320	no	-	-
279	***RNU6-449P***	15:54056401-54056507	no	-	-
280	***UNC13C***	15:54270377-54920806	yes	614568	May play a role in vesicle maturation during exocytosis as a target of the diacylglycerol second messenger pathway. May be involved in the regulation of synaptic transmission at parallel fiber - Purkinje cell synapses (By similarity).
281	***LOC100422032***	15:54340181-54343168	no	-	-
282	***HNRNPA1P74***	15:54612736-54613930	no	-	-
283	***LOC105370829***	15:55348945-55384371	no	-	-
284	***RSL24D1***	15:55473512-55489231	yes	613262	Involved in the biogenesis of the 60S ribosomal subunit. Ensures the docking of GTPBP4/NOG1 to pre-60S particles (By similarity).
285	***RAB27A***	15:55495164-55582013	yes	603868	Small GTPase which cycles between active GTP-bound and inactive GDP-bound states. In its active state, binds to a variety of effector proteins to regulate homeostasis of late endocytic pathway,

					including endosomal positioning, maturation and secretion (PubMed:30771381). Plays a role in cytotoxic granule exocytosis in lymphocytes. Required for both granule maturation and granule docking and priming at the immunologic synapse.
286	***LOC100506294***	15:55609382-55611392	no	-	-
287	***PIGB***	15:55611133-55647846	yes	604122	Mannosyltransferase involved in glycosylphosphatidylinositol-anchor biosynthesis. Transfers the third alpha-1,2-mannose to Man2-GlcN-acyl-PI during GPI precursor assembly.
288	***CCPG1***	15:55647421-55700708	yes	611326	Acts as an assembly platform for Rho protein signalling complexes. Limits guanine nucleotide exchange activity of MCF2L toward RHOA, which results in an inhibition of both its transcriptional activation ability and its transforming activity. Does not inhibit activity of MCF2L toward CDC42, or activity of MCF2 toward either RHOA or CDC42 (By similarity). May be involved in cell cycle regulation.
289	***DNAAF4-CCPG1***	15:55647421-55790782	no	-	-
290	***MIR628***	15:55665138-55665232	no	-	-
291	***C15orf65***	15:55700723-55710910	yes	-	PIERCE2 (Piercer Of Microtubule Wall 2) is a Protein Coding gene. An important paralog of this gene is PIERCE1.Predicted to act upstream of or within several processes, including cellular response to DNA damage stimulus; cellular

					response to UV-C; and determination of left/right symmetry. Predicted to be located in nucleus. [provided by Alliance of Genome Resources, Apr 2022]
292	***DNAAF4***	15:55709953-55800432	yes	608706	Axonemal dynein assembly factor required for ciliary motility. Involved in neuronal migration during development of the cerebral neocortex. May regulate the stability and proteasomal degradation of the estrogen receptors that play an important role in neuronal differentiation, survival and plasticity.
293	***LOC100420711***	15:55734633-55735623	no	-	-
294	***PYGO1***	15:55831079-55881050	yes	606902	Involved in signal transduction through the Wnt pathway.
295	***PRTG***	15:55903738-56035317	yes	613261	May play a role in anteroposterior axis elongation.
296	***NEDD4***	15:56119115-56285944	yes	602278	(Microbial infection) Involved in the ubiquitination of Ebola virus protein VP40 which plays a role in viral budding. E3 ubiquitin-protein ligase which accepts ubiquitin from an E2 ubiquitin-conjugating enzyme in the form of a thioester and then directly transfers the ubiquitin to targeted substrates. Specifically, ubiquitinates 'Lys-63' in target proteins (PubMed:23644597). Involved in the pathway leading to the degradation of VEGFR-2/KDFR, independently of its ubiquitin-ligase activity. Monoubiquitinates IGF1R at multiple sites, thus leading to receptor internalization and degradation

					in lysosomes. Ubiquitinates FGFR1, leading to receptor internalization and degradation in lysosomes. Promotes ubiquitination of RAPGEF2. According to PubMed:18562292 the direct link between NEDD4 and PTEN regulation through polyubiquitination described in PubMed:17218260 is questionable. Involved in ubiquitination of ERBB4 intracellular domain E4ICD. Involved in the budding of many viruses. Part of a signalling complex composed of NEDD4, RAP2A and TNIK which regulates neuronal dendrite extension and arborization during development. Ubiquitinates TNK2 and regulates EGF-induced degradation of EGFR and TNF2. Ubiquitinates BRAT1 and this ubiquitination is enhanced in the presence of NDFIP1 (PubMed:25631046). Ubiquitinates DAZAP2, leading to its proteasomal degradation (PubMed:11342538).
297	***CNOT6LP1***	15:56295503-56299377	no	-	-
298	***RN7SL568P***	15:56321882-56322179	no	-	-
299	***CD24P2***	15:56363537-56366286	no	-	-
300	***RFX7***	15:56382731-56535483	yes	612660	RFX7 is a member of the regulatory factor X (RFX) family of transcription factors (see RFX1, MIM 600006) (Aftab et al., 2008 [PubMed 18673564]).
301	***LOC390586***	15:56483782-56485548	no	-	-
302	***RNU6-1287P***	15:56566728-56566834	no	-	-

303	***HMGB1P33***	15:56615207-56615935	no	-	-
304	***TEX9***	15:56657622-56738195	yes	-	TEX9 (Testis Expressed 9) is a Protein Coding gene. Diseases associated with TEX9 include Heterotaxy, Visceral, 9, Autosomal, With Male Infertility and Dextrocardia with Situs Inversus.
305	***MNS1***	15:56720929-56757335	yes	610766	This gene encodes a protein highly similar to the mouse meiosis-specific nuclear structural 1 protein. The mouse protein was shown to be expressed at the pachytene stage during spermatogenesis and may function as a nuclear skeletal protein to regulate nuclear morphology during meiosis. [provided by RefSeq, Oct 2008]
306	***ZNF280D***	15:56922374-57026284	yes	-	Predicted to enable DNA-binding transcription factor activity, RNA polymerase II-specific and RNA polymerase II cis-regulatory region sequence-specific DNA binding activity. Predicted to be involved in regulation of transcription, DNA-templated. Predicted to be located in nucleus. [provided by Alliance of Genome Resources, Apr 2022]
307	***LOC645877***	15:57138511-57139046	no	-	-
308	***LOC145783***	15:57178368-57210697	no	-	-
309	***TCF12***	15:57210288-57583459	yes	600480	The protein encoded by this gene is a member of the basic helix-loop-helix (bHLH) E-protein family that recognizes the consensus binding site (E-box)

					CANNTG. This encoded protein is expressed in many tissues, among them skeletal muscle, thymus, B- and T-cells, and may participate in regulating lineage-specific gene expression through the formation of heterodimers with other bHLH E-proteins. Several alternatively spliced transcript variants of this gene have been described, but the full-length nature of some of these variants has not been determined. [provided by RefSeq, Jul 2008

Table S2 List of variants in 1 % control database overlapping OMIM genes.

No.	Chromosome	Type	Refstart-Refend(bp)	Gene (OMIM)	Function (NCBI Entrez Gene)
1	1	deletion	117,591,182- 117,600,131	-	-
2	2	deletion	80,163,783-80,177,072.5	***CTNNA2***(114025)	Enables actin filament binding activity. Involved in negative regulation of Arp2/3 complex-mediated actin nucleation; regulation of neuron migration; and regulation of neuron projection development. Located in cytoplasm. Implicated in complex cortical dysplasia with other brain malformations.
3	22	deletion	38,353,165.6-38,359,400.4	***POLR2F*** (604414)	This gene encodes the sixth largest subunit of RNA polymerase II, the polymerase responsible for

					synthesizing messenger RNA in eukaryotes. In yeast, this polymerase subunit, in combination with at least two other subunits, forms a structure that stabilizes the transcribing polymerase on the DNA template.
4	23	deletion	69,022,600-69,027,051	***EDA*** (300451)	The protein encoded by this gene is a type II membrane protein that can be cleaved by furin to produce a secreted form. The encoded protein, which belongs to the tumor necrosis factor family, acts as a homotrimer and may be involved in cell-cell signaling during the development of ectodermal organs. Defects in this gene are a cause of ectodermal dysplasia, anhidrotic, which is also known as X-linked hypohidrotic ectodermal dysplasia.
5	1	insertion	111,664,385.8-111,665,594.2	***DRAM2*** (613360)	The protein encoded by this gene binds microtubule-associated protein 1 light chain 3 and is required for autophagy. Defects in this gene are a cause of retinal dystrophy. In addition, two microRNAs (microRNA 125b-1 and microRNA 144) can bind to the mRNA of this gene and produce the disease

					state.
6	16	insertion	28,656,660-28,656,660	***NPIPL1***	**-**
7	16	insertion	77,457,768-77,459,504	***ADAMTS18***(613360)	This gene encodes a member of the ADAMTS (a disintegrin and metalloproteinase with thrombospondin motifs) protein family. ADAMTS family members share several distinct protein modules, including a propeptide region, a metalloproteinase domain, a disintegrin-like domain, and a thrombospondin type 1 (TS) motif. Individual members of this family differ in the number of C-terminal TS motifs, and some have unique C-terminal domains. The encoded preproprotein is proteolytically processed to generate the mature protein, which may regulate hemostatic balance and function as a tumor suppressor. Mutations in this gene may be associated with microcornea, myopic chorioretinal atrophy, and telecanthus (MMCAT) and cone-rod dystrophy in human patients.

8	22	insertion	23,439,875-23,475,792	***RTDR1*** (605663)	This gene encodes a protein with no known function but with slight similarity to a yeast vacuolar protein. The gene is located in a region deleted in pediatric rhabdoid tumors of the brain, kidney and soft tissues, but mutations in this gene have not been associated with the disease.
				GNAZ (139160)	The protein encoded by this gene is a member of a G protein subfamily that mediates signal transduction in pertussis toxin-insensitive systms. This encoded protein may play a role in maintaining the ionic balance of perilymphatic and endolymphatic cochlear fluids.
9	6	insertion	45,918,709-45,934,658	***CLIC5*** (607293)	This gene encodes a member of the chloride intracellular channel (CLIC) family of chloride ion channels. The encoded protein associates with actin-based cytoskeletal structures and may play a role in multiple processes including hair cell stereocilia formation, myoblast proliferation and glomerular podocyte and endothelial cell maintenance. Alternatively spliced

					transcript variants encoding multiple isoforms have been observed for this gene.
10	16	duplication	29,459,257-29,509,729	***LOC606724***	**-**
				BOLA2 (613182)	This gene is located within a region of a segmental duplication on chromosome 16 and is identical to BOLA2B (bolA family member 2B). The product of this gene belongs to a family of proteins that are widely conserved and may be involved in iron maturation. Related pseudogenes are found multiple different chromosomes. Alternative splicing results in multiple transcript variants. Transcripts initiating at this locus may extend into downstream SMG1 pseudogene 6 (SMG1P6) and encode fusion proteins with a C-terminus related to SMG1 phosphatidylinositol 3-kinase-related kinase.
				SLX1A (615822)	This gene encodes a protein that is an important regulator of genome stability. The protein represents the catalytic subunit of the SLX1-SLX4 structure-specific endonuclease, which can

					resolve DNA secondary structures that are formed during repair and recombination processes. Two identical copies of this gene are located on the p arm of chromosome 16 due to a segmental duplication; this record represents the more centromeric copy. Alternative splicing results in multiple transcript variants. Read-through transcription also occurs between this gene and the downstream SULT1A3 (sulfotransferase family, cytosolic, 1A, phenol-preferring, member 3) gene.
				SULT1A3 (600641)	Sulfotransferase enzymes catalyze the sulfate conjugation of many hormones, neurotransmitters, drugs, and xenobiotic compounds. These cytosolic enzymes are different in their tissue distributions and substrate specificities. The gene structure (number and length of exons) is similar among family members. This gene encodes a phenol sulfotransferase with thermolabile enzyme activity. Four sulfotransferase genes are located on the p arm of chromosome 16; this gene and

					SULT1A4 arose from a segmental duplication. This gene is the most centromeric of the four sulfotransferase genes. Read-through transcription exists between this gene and the upstream SLX1A (SLX1 structure-specific endonuclease subunit homolog A) gene that encodes a protein containing GIY-YIG domains.
				LOC613038	**-**
				LOC100132247	**-**
				LOC23117	**-**
				DQ576952	**-**
				NPIPL3	**-**
11	16	duplication	75,549,007.5-75,577,384	***CHST5*** (604817)	The protein encoded by this gene belongs to the Gal/GalNAc/GlcNAc 6-O-sulfotransferase (GST) family, members of which catalyze the transfer of sulfate to position 6 of galactose (Gal), N-acetylgalactosamine (GalNAc), or N-acetylglucosamine (GlcNAc) residues within proteoglycans, and sulfation of O-linked sugars of mucin-type acceptors. Carbohydrate sulfation plays a critical role in many biologic processes. This

					gene is predominantly expressed in colon and small intestine.
				TMEM231(614949)	This gene encodes a transmembrane protein, which is a component of the B9 complex involved in the formation of the diffusion barrier between the cilia and plasma membrane. Mutations in this gene cause Joubert syndrome (JBTS). Multiple alternatively spliced transcript variants have been found for this gene.
12	X	duplication	7,706,472-7,997,402	***VCX*** (300229)	This gene belongs to the VCX/Y gene family, which has multiple members on both X and Y chromosomes, and all are expressed exclusively in male germ cells. The X-linked members are clustered on chromosome Xp22 and Y-linked members are two identical copies of the gene within a palindromic region on Yq11. The family members share a high degree of sequence identity, with the exception that a 30-bp unit is tandemly repeated in X-linked members but occurs only once in Y-linked members. The VCX gene cluster is polymorphic in terms of copy

					number; different individuals may have a different number of VCX genes. VCX/Y genes encode small and highly charged proteins of unknown function. The presence of a putative bipartite nuclear localization signal suggests that VCX/Y members are nuclear proteins.
				PNPLA4 (300102)	This gene encodes a member of the patatin-like family of phospholipases. The encoded enzyme has both triacylglycerol lipase and transacylase activities and may be involved in adipocyte triglyceride homeostasis. Alternate splicing results in multiple transcript variants. A pseudogene of this gene is found on chromosome Y.
13	Y	duplication _ inverted	25,415,297-25,427,212	***DAZ2*** (400026)	This gene is a member of the DAZ gene family and is a candidate for the human Y-chromosomal azoospermia factor (AZF). Its expression is restricted to premeiotic germ cells, particularly in spermatogonia. It encodes an RNA-binding protein that is important for spermatogenesis. Four copies of this gene are found on chromosome Y within palindromic duplications; one

					pair of genes is part of the P2 palindrome and the second pair is part of the P1 palindrome. Each gene contains a 2.4 kb repeat including a 72-bp exon, called the DAZ repeat; the number of DAZ repeats is variable and there are several variations in the sequence of the DAZ repeat. Each copy of the gene also contains a 10.8 kb region that may be amplified; this region includes five exons that encode an RNA recognition motif (RRM) domain. This gene contains one copy of the 10.8 kb repeat. Alternative splicing results in multiple transcript variants encoding different isoforms.

Figure S1. Top 20 clusters of GO enrichment for the genes in duplication of 15q14q21.3.

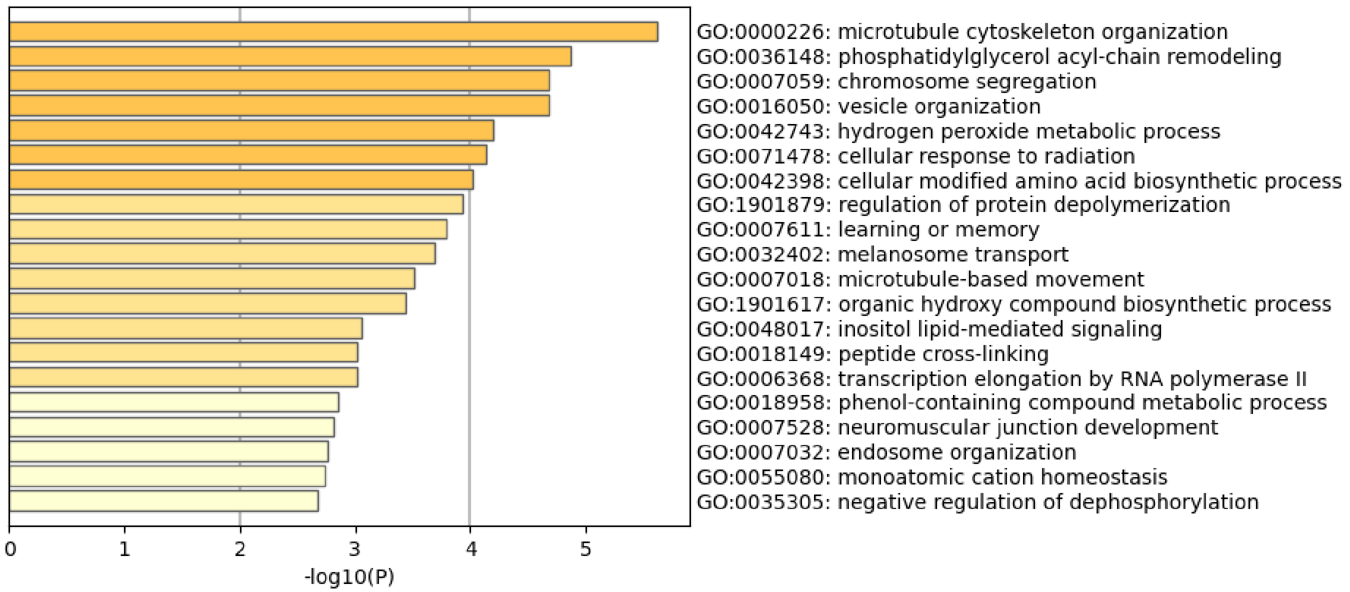

Figure S2. Top 3 clusters of KEGG pathway enrichment for the genes in duplication of 15q14q21.3.

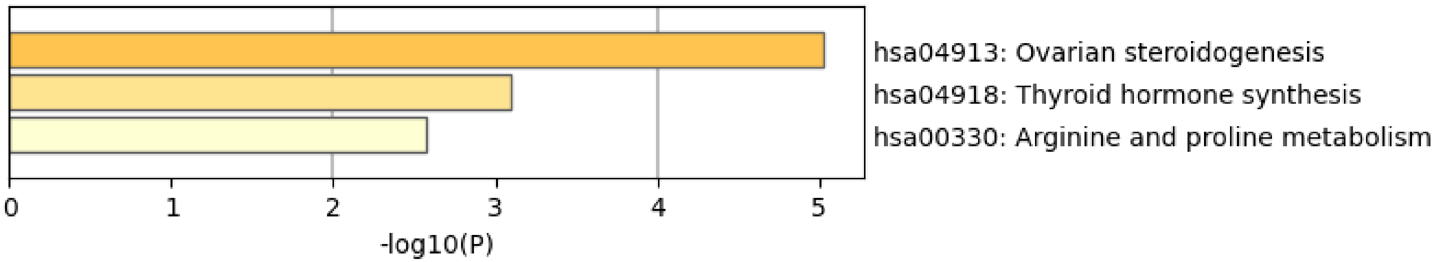

Figure S3. Results of Sanger sequencing for two breakpoints. (a) and (b) breakpoints one;(c) and (d) breakpoints two.

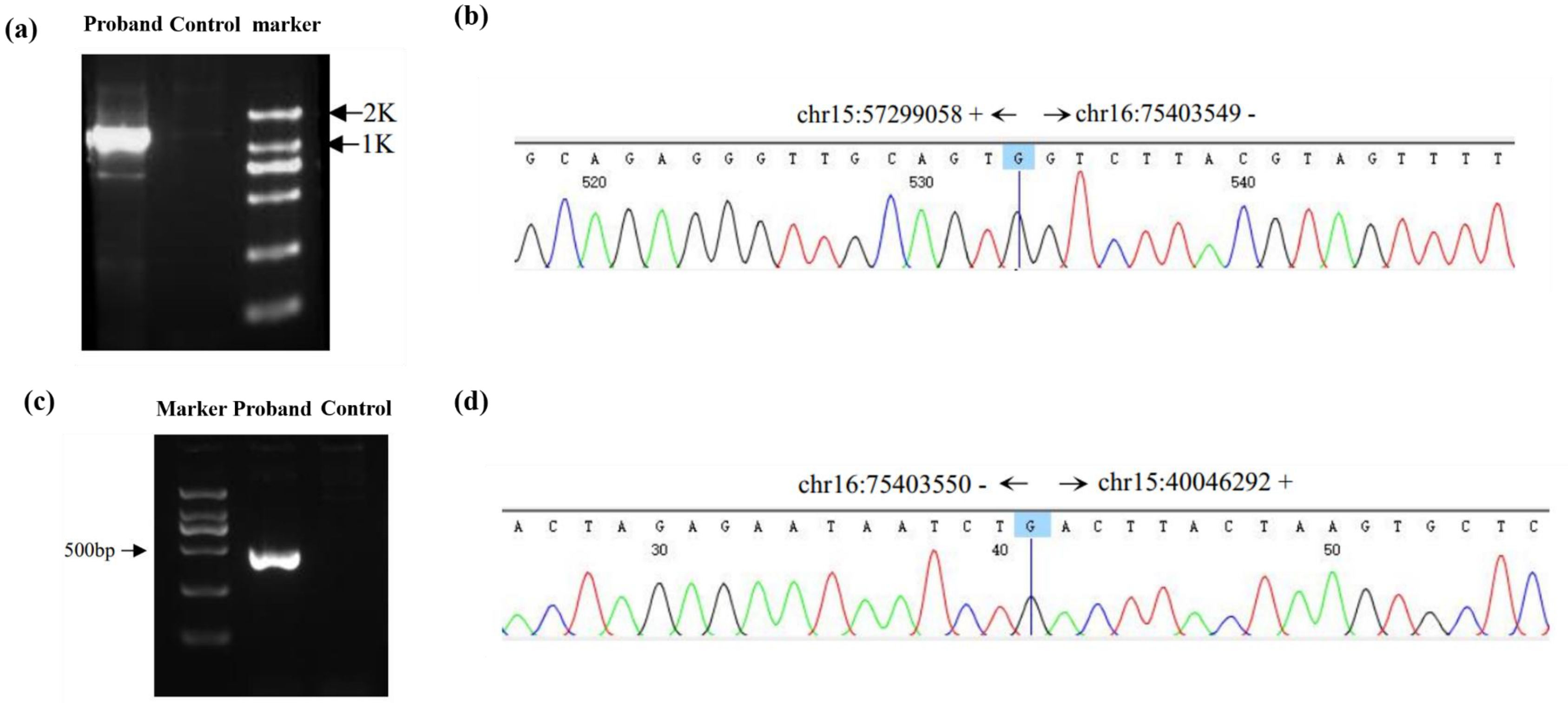

Supplement: Supplementary file 1 [file genes-14-01016-s001.zip › genes-2340511-supplementary.pdf]
